# Physiologically Based Pharmacokinetic Modeling of Monoclonal Antibodies in Pediatric Populations Using PK-Sim

**DOI:** 10.3389/fphar.2020.00868

**Published:** 2020-06-11

**Authors:** Sumit Basu, Yi Ting (Kayla) Lien, Valvanera Vozmediano, Jan-Frederik Schlender, Thomas Eissing, Stephan Schmidt, Christoph Niederalt

**Affiliations:** ^1^Center for Pharmacometrics and Systems Pharmacology, College of Pharmacy, University of Florida, Orlando, FL, United States; ^2^Clinical Pharmacometrics, Bayer AG, Leverkusen, Germany

**Keywords:** physiologically based pharmacokinetic (PBPK) modeling, PK-Sim, monoclonal antibodies, pediatrics, children

## Abstract

Physiologically based pharmacokinetic (PBPK) models are increasingly used to support pediatric dose selection for small molecule drugs. In contrast, only a few pediatric PBPK models for therapeutic antibodies have been published recently, and the knowledge on the maturation of the processes relevant for antibody pharmacokinetics (PK) is limited compared to small molecules. The aim of this study was, thus, to evaluate predictions from antibody PBPK models for children which were scaled from PBPK models for adults in order to identify respective knowledge gaps. For this, we used the generic PBPK model implemented in PK-Sim without further modifications. Focusing on general clearance and distribution mechanisms, we selected palivizumab and bevacizumab as examples for this evaluation since they show simple, linear PK which is not governed by drug-specific target mediated disposition at usual therapeutic dosages, and their PK has been studied in pediatric populations after intravenous application. The evaluation showed that the PK of palivizumab was overall reasonably well predicted, while the clearance for bevacizumab seems to be underestimated. Without implementing additional ontogeny for antibody PK-specific processes into the PBPK model, bodyweight normalized clearance increases only moderately in young children compared to adults. If growth during aging at the time of the simulation was considered, the apparent clearance is approximately 20% higher compared to simulations for which growth was not considered for newborns due to the long half-life of antibodies. To fully understand the differences and similarities in the PK of antibodies between adults and children, further research is needed. By integrating available information and data, PBPK modeling can contribute to reveal the relevance of involved processes as well as to generate and test hypothesis.

## Introduction

Pediatric drug development has accelerated over the last two decades due to increased regulatory requirements in response to unmet medical needs in this special patient population (Manolis et al., 2011; Leong et al., 2012). However, drug treatment of pediatrics is in many cases still empirical and characterized by high off-label use. This is due to the fact that there is a lack of clinical data in pediatrics, especially in infants and neonates. As a result, there is uncertainty about the impact of growth and maturation on pediatric pharmacokinetics (PK) and pharmacodynamics (PD) and, thus, dose (Samant et al., 2015). Therefore, dose-selection for pediatrics has to frequently rely on available adult clinical data as the basis for extrapolation. Approaches used for dose extrapolation can vary greatly in their complexity ranging from simple allometric scaling approaches to more complex physiologically based scaling approaches. However, if there are clinically relevant differences in disease progression and/or response to therapeutic interventions, extrapolation of adult doses may not be feasible and require the conduct of pediatric efficacy and safety trials. In cases where extrapolation is feasible, allometric scaling is usually the standard choice for small molecules for children 2 years and older. Additional maturational processes, particularly phase I and phase II enzyme ontogeny, may have to be considered in children younger than two years of age for small molecule drugs. This has given rise to more complex approaches, including physiologically based pharmacokinetic (PBPK) models for which there is meanwhile considerable experience with scaling PBPK models to pediatric populations for small molecule drugs (Leong et al., 2012; Templeton et al., 2018; Heimbach et al., 2019; Willmann et al., 2019). For therapeutic proteins like antibodies, however, there is currently only very limited experience with scaling PBPK models to children (Hardiansyah and Ng, 2018; Hanke et al., 2019; Malik and Edginton, 2019; Malik and Edginton, 2020), and there is currently only limited quantitative information regarding the maturation of the processes relevant for antibody PK (Shi and Derendorf, 2010; Xu et al., 2013; Edlund et al., 2015; Zhang et al., 2015; Liu et al., 2019).

Therefore, the objective of our study was to evaluate the predictive performance of pediatric PBPK models for antibodies using selected case examples without adding any additional ontogeny for processes specific to antibodies.

## Methods

### PK Data

**Figure 1:**
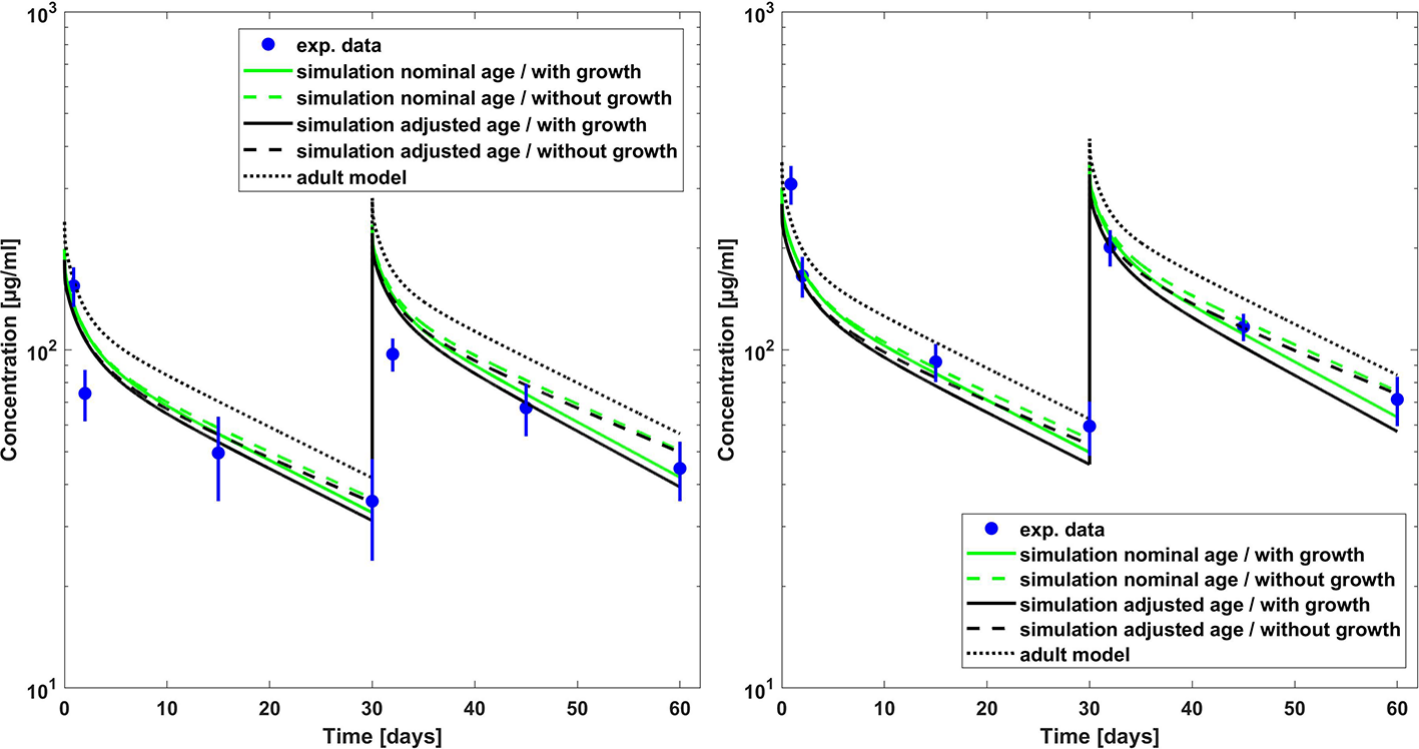
Comparison of PBPK model predicted (lines) versus experimental (symbols) plasma concentration–time profiles of palivizumab in children for a dose of 10 mg/kg (left hand side) and 15 mg/kg (right hand side). Predictions from the adult model are shown for comparison. Experimental data (mean concentrations and standard error) are taken from a study which included children which had bronchopulmonary dysplasia or which were born premature (Subramanian et al., 1998).

**Figure 2:**
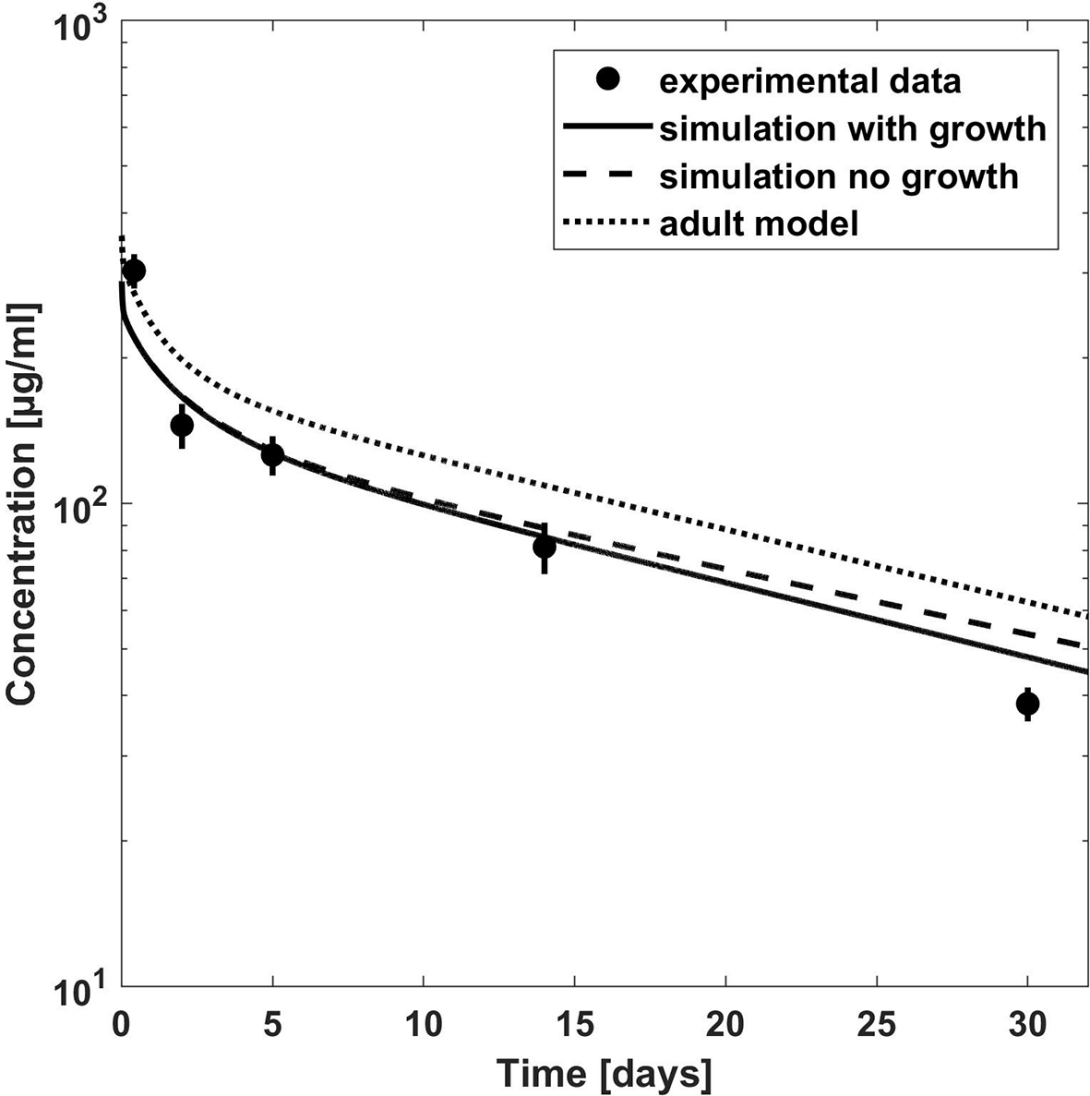
Comparison of PBPK model predicted (lines) versus experimental (symbols) plasma concentration–time profiles of palivizumab in children for a dose 15 mg/kg. Predictions from the adult model are shown for comparison. Experimental data (mean concentrations and standard error) are taken from a study which included children with RSV infection (Saez-Llorens et al., 2004).

**Figure 3:**
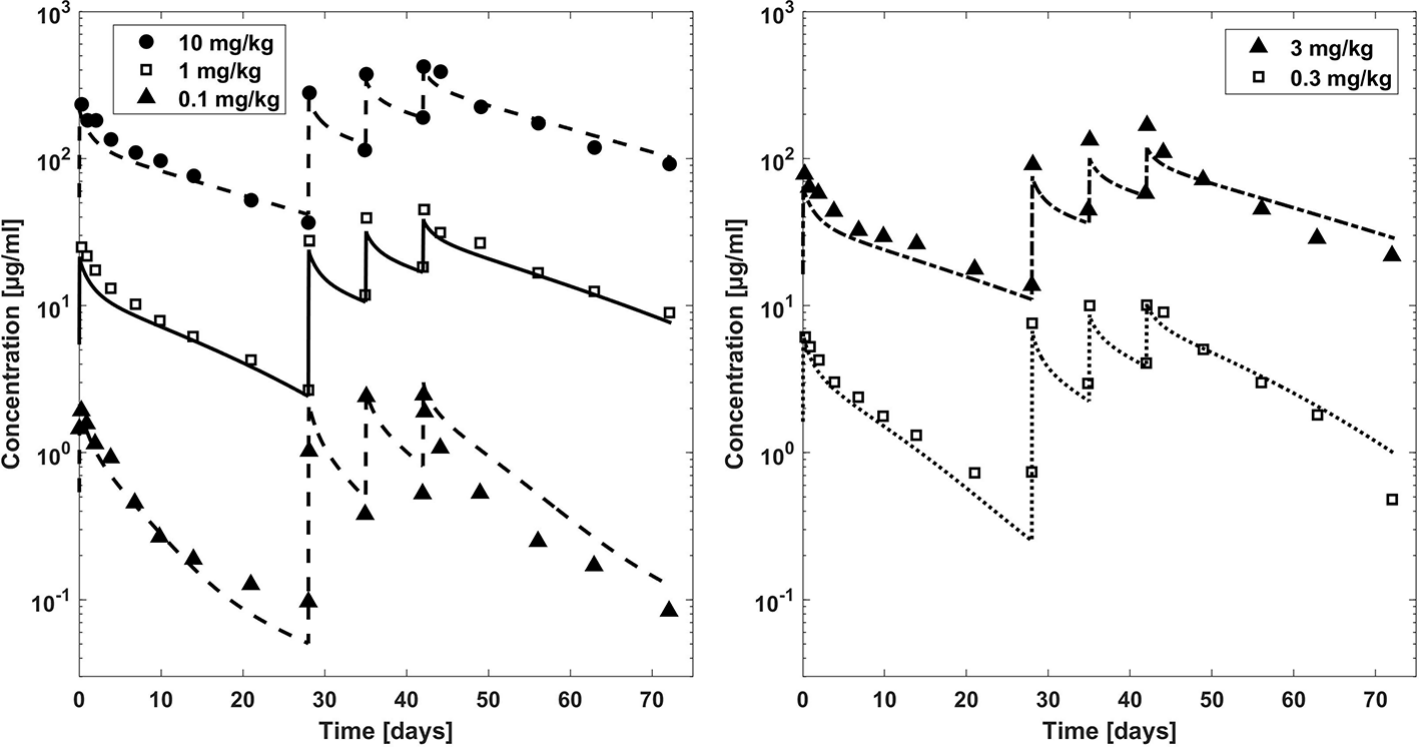
Comparison plasma concentration–time profiles of bevacizumab fitted with the PBPK model (lines) versus experimental data (symbols) for adults after a dose of 0.1, 1, and 10 mg/kg (left hand side) and 0.3 and 3 mg/kg (right hand side). Experimental data (mean concentrations) are taken from literature (Gordon et al., 2001).

**Figure 4:**
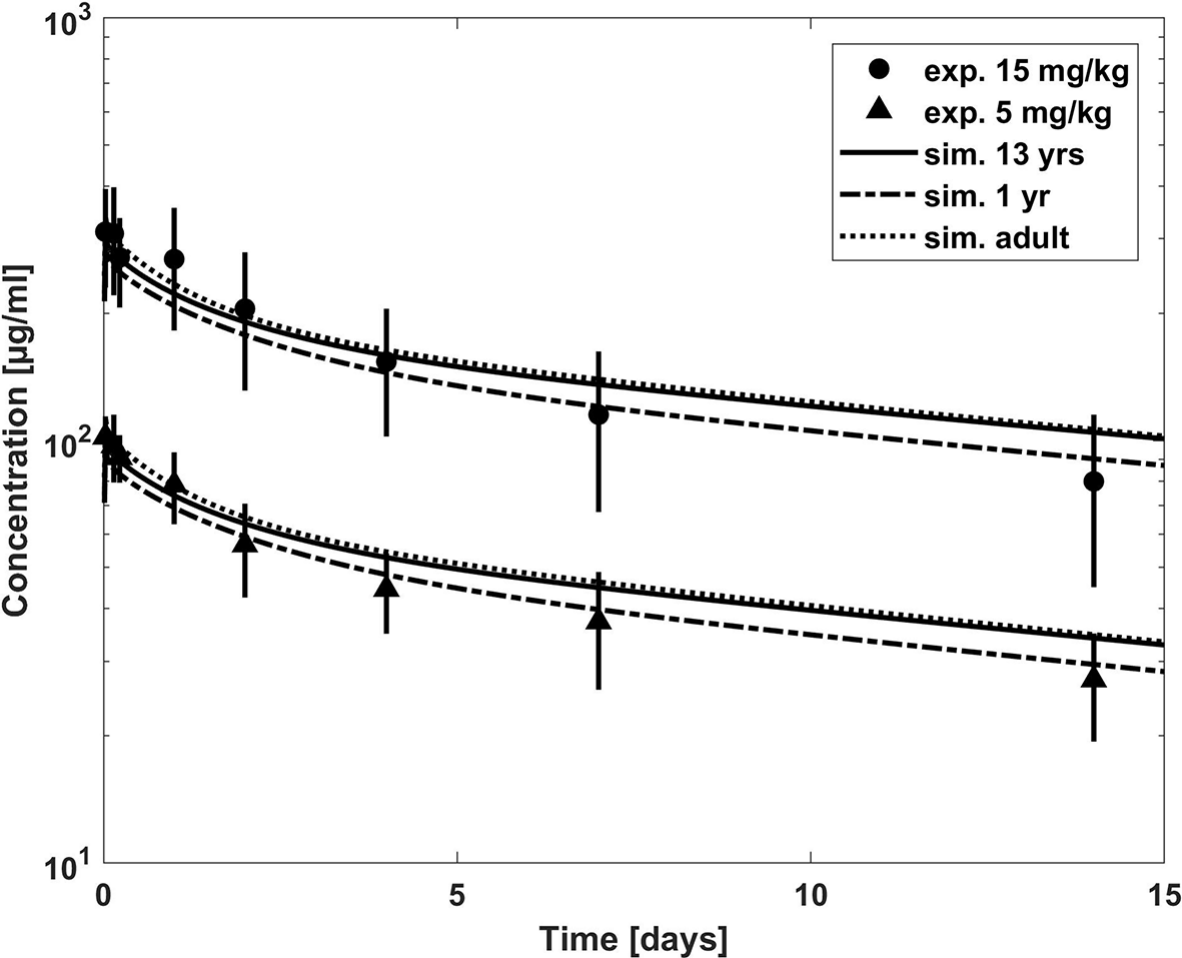
Comparison of concentration–time profiles of bevacizumab predicted with the PBPK model for children (with growth) and adult (lines) versus experimental data (symbols) after a dose of 5 and 15 mg/kg. The experimental data represent mean concentrations from a population with an age range from 1 to 21 years; median age, 13 years (Glade Bender et al., 2008).

**Figure 5:**
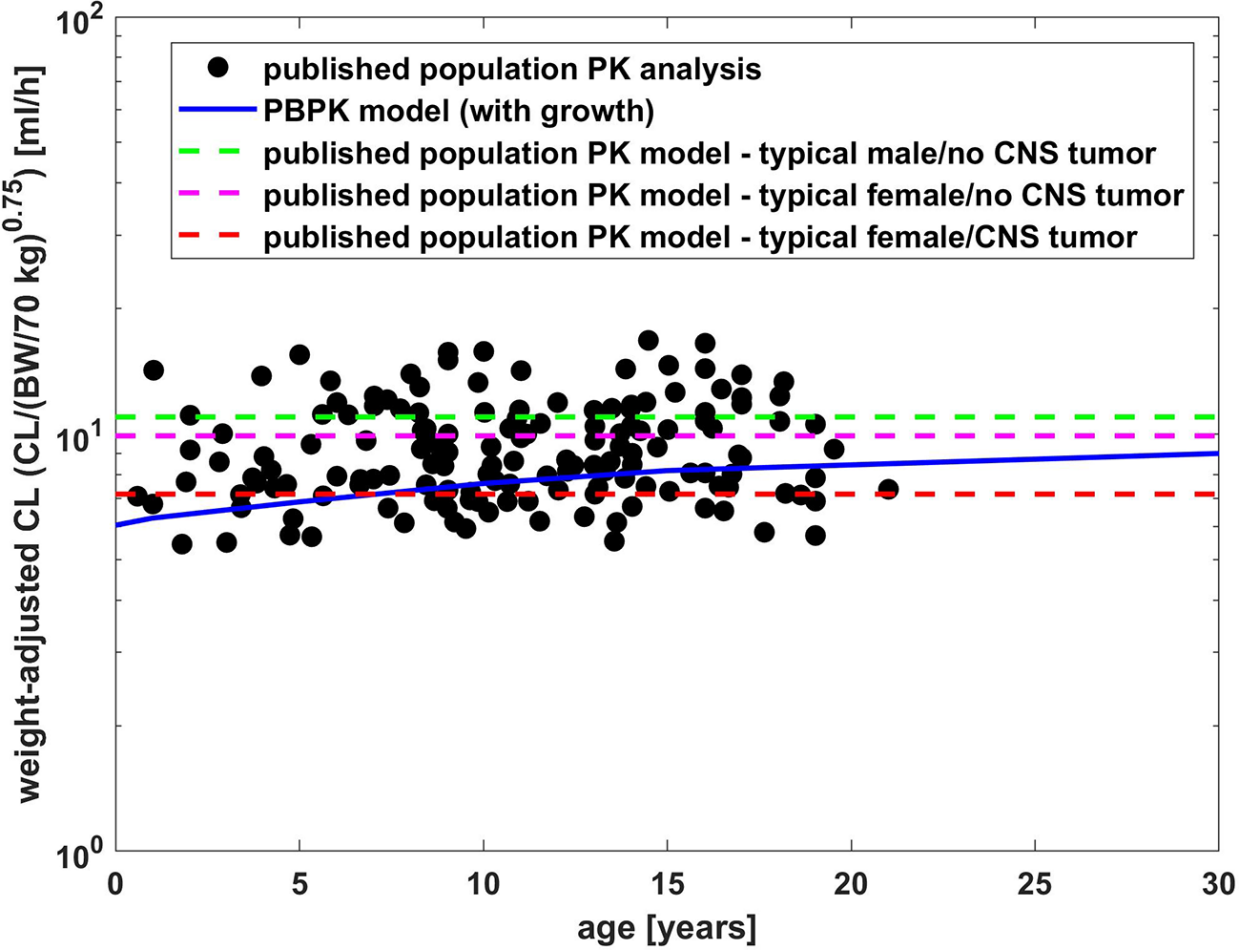
Comparison of weight adjusted clearance [CL/(body weight/70 kg)^0.75^] across different ages simulated by PBPK model of bevacizumab and estimated from a population PK analysis (Han et al., 2016b).

We selected palivizumab and bevacizumab, as examples for the evaluation since they show linear PK, which is not governed by target mediated drug disposition (TMDD) at usual therapeutic dosages and are applied intravenously. Only intravenous dosage forms of biologics were considered to avoid additional absorption- and formulation-related complexities present after subcutaneous and intramuscular application leading to a reduction of bioavailability and a slow lymphatic absorption. These selection criteria allow us to focus on the evaluation of the generic PK-Sim model with respect to distribution and nonspecific clearance in children. The PK of palivizumab and bevacizumab has been extensively studied (Subramanian et al., 1998; Gordon et al., 2001; Saez-Llorens et al., 2004; Glade Bender et al., 2008; Robbie et al., 2012; Han et al., 2016b) covering an age range from newborn to 24 months for palivizumab and of 0.5 to 21 years for bevacizumab (cf. **Table 1**). Palivizumab is a humanized IgG1 monoclonal antibody directed against the F-protein of the respiratory syncytial virus (RSV) (Robbie et al., 2012) with a plasma half-life of 17 to 26.8 days (Subramanian et al., 1998; Saez-Llorens et al., 2004). Bevacizumab is a humanized IgG1 monoclonal antibody that binds to the human vascular endothelial growth factor (VEGF) (Wang et al., 2004) and has a plasma half-life of ~ 20 days for dosages of 1 to 20 mg/kg (Han et al., 2016a). The data shown in **Figures 1**–**5** have been digitized from the published figures with the in-house tool ChartScanner.

**Table 1:** List of studies used for the development and qualification of the PBPK models in the current study.

Drug	Target	Age (Pediatric Population)	Number of individuals	Description and References
Palivizumab	RSV Protein F	Adults and children younger than 24 months	1,883(1756 children)	Population PK analysis using data from 22 clinical studies (Robbie et al., 2012)
		6.9 ± 1.3 months (3 mg/kg)^#^	10^#^	Phase I/II study in premature infants and infants with bronchopulmonary dysplasia (Subramanian et al., 1998)
		7.39 ± 2 months (10 mg/kg)	10	
		8.19 ± 1.7 months (15 mg/kg)	22	
		1.5 ± 0.4 months (5 mg/kg)^#^	8^#^	Phase I/II study in children with RSV infection (Saez-Llorens et al., 2004)
		5.2 ± 0.9 months (15 mg/kg)	22	
Bevacizumab	VEGF-A	Adults	25 in 5 dose groups	Phase I study in cancer patients (Gordon et al., 2001)
		1–21 years, median, 13 years (total study)	20 total, 8 PK	Phase I study in pediatric cancer patients (Glade Bender et al., 2008)
		0.5–21 years, median, 10.8 years	152 for model building	Population PK analysis using data from cancer patients (5 studies) (Han et al., 2016b)

^#^For the low dosages, only the reported PK parameters were used for comparison but not the concentration-time profiles, since they could not be read with sufficient accuracy from the published figure.

Besides comparison to experimental plasma concentration-time profiles, the PBPK results were additionally compared to simulated PK data from 2-compartment models from population PK analyses which have been published for palivizumab and bevacizumab in pediatric populations (Robbie et al., 2012; Han et al., 2016b). For palivizumab, the parameters of the 2-compartment model were given by the following equations (Robbie et al., 2012):

$$Cl=198\;ml/day\cdot {\left(\frac{BW\;\left(kg\right)}{70\;kg}\right)}^{0.75}\cdot \left(1-\left(1-0.411\right)\cdot e^{\left(-\left(\frac{PAGE-40}{4.35}\right)\cdot \frac{\text{ln}\left(2\right)}{62.3}\right)}\right)\;$$

$$V_{c}=4.09\;L\cdot {\left(\frac{BW\;\left(kg\right)}{70\;kg}\right)}^{1}\;$$

$$V_{p}=2.23\;L\cdot {\left(\frac{BW\;\left(kg\right)}{70\;kg}\right)}^{1}\;$$

$$Q=879\;ml/day\cdot {\left(\frac{BW\;\left(kg\right)}{70\;kg}\right)}^{0.75}$$

where Cl represents clearance, BW represents body weight, PAGE represents the sum of gestational age and postnatal age in weeks, Vc represents the volume of the central compartment, Vp represents the volume of the peripheral compartment, and Q represents the inter-compartmental clearance. In order to describe the clearance of palivizumab, an age-dependent maturation factor was used additionally to the allometric scaling with an exponent of 0.75. Note that the equation for the maturation factor was taken from the publication by Anderson et al. (2006) referenced in the erratum of the population PK analysis (Robbie et al., 2012) and is in agreement with the values for the reference child reported in Table 3 of the publication reporting the population PK analysis (Robbie et al., 2012). The increase of body weight during simulation time was considered for children younger than 4 years in the same way as for missing body weight records in the population PK study (Robbie et al., 2012):

$$If\;PAGE_{i}<13\;\text{months}:BW_{i}=BW_{i-1}+0.89\cdot \left(PAGE_{i}-PAGE_{i-1}\right)$$

$$\begin{array}{l} If\;PAGE_{i}\;\geq 13\;\text{months }and\;PAGE_{i-1}\geq 13\;\text{months}:\\ BW_{i}=BW_{i-1}+0.24\cdot \left(PAGE_{i}-PAGE_{i-1}\right) \end{array}$$

$$\begin{array}{l} If\;PAGE_{i}\;\geq 13\;\text{months }and\;PAGE_{i-1}<13\;\text{months }:\\ BW_{i}=BW_{i-1}+0.89\cdot \left(13-PAGE_{i-1}\right)+0.24\cdot \left(PAGE_{i}-13\right) \end{array}$$

For bevacizumab, the parameters of the published 2-compartment model (Han et al., 2016b) were given by:

$$Cl=237.6\frac{ml}{day}\cdot {\left(\frac{BW\;\left(kg\right)}{70\;kg}\right)}^{0.75}\cdot 1.11\;\left(\text{if male}\right)\cdot 0.725\;\left(\text{if primary CNS tumors}\right)$$

$$V_{c}=2.850\;L\cdot {\left(\frac{BW\;\left(kg\right)}{70\;kg}\right)}^{0.701}\;\cdot 1.14\;\left(\text{if male}\right)\cdot 0.854\;\left(\text{if primary CNS tumors}\right)$$

$$V_{p}=2.564\;L\cdot {\left(\frac{BW\;\left(kg\right)}{70\;kg}\right)}^{0.766}\;$$

$$Q=672\;ml/day\cdot {\left(\frac{BW\;\left(kg\right)}{70\;kg}\right)}^{0.75}$$

If available, we compared the PBPK predictions to measured plasma concentration-time profiles. Additionally we compared PBPK prediction to the primary PK parameters clearance (CL) and volume of distribution at steady state (Vss), as well as the secondary PK parameters “area under the plasma concentration-time curve” (AUC) and the terminal half-life (T_1/2_) reported in literature or calculated from a non-compartmental analysis (NCA) of plasma concentration-time profiles simulated from the published 2-compartment models used in population PK analyses (population PK models). The PK parameters for the PBPK models were also calculated by a NCA analysis of the simulated plasma concentration-time profiles.

### Software Used

All PBPK models (adult and pediatric) were built using the Open Systems Pharmacology Suite (OSP Suite, http://www.open-systemspharmacology.org), version 8.0. The suite comprises the PBPK software tool PK-Sim and the modeling software tool MoBi, which was used to extend the PBPK models with additional specific processes related to TMDD. MATLAB (R2017b; The MathWorks, Inc., Natick, Massachusetts) was used to perform PBPK simulations and to generate plots *via* the MATLAB-Toolbox of the OSP Suite.

### PBPK Model Structure

The PBPK model for therapeutic proteins and macromolecules in PK-Sim including the corresponding generic systems parameters (Niederalt et al., 2018) was used to build the antibody PBPK models. Briefly, the PBPK model in PK-Sim contains 15 organs or tissues. The model for therapeutic proteins represents an extension of the small molecule PBPK model which considers additionally passive exchange across the vascular endothelium, return of drug *via* lymph flow to the systemic circulation, drug catabolism in the endosomal space, and protection from catabolism by FcRn. Distribution of therapeutic proteins is governed by transcapillary exchange of the protein between plasma and interstitial spaces. Transcapillary exchange is represented by the two-pore formalism, which describes the barrier between plasma and interstitial space as a membrane consisting of two different sized types of pores (Rippe and Haraldsson, 1987; Rippe and Haraldsson, 1994). Proteins can pass through these pores by two different mechanisms, namely, convection and diffusion. Different drug- and system-specific parameters, such as hydrodynamic radius of the drug, endothelial pore radii (both small and large), fraction of flow *via* large pores, and hydraulic conductivity of the vascular endothelium influence both convection and diffusion rates.

While the drugs selected for the current analysis do not show TMDD at therapeutic dosages applied to children, the bevacizumab data for adults reported by Gordon et al. (Gordon et al., 2001) additionally included sub-therapeutic dosages showing TMDD. In order to use all reported dose steps for the model development for bevacizumab in adults, we extended the general PK-Sim model by TMDD processes to define an additional saturable clearance mechanism for general model applicability. However, this process has only negligible effect within the pediatric therapeutic dose range assuming no maturation of target concentration (cf. **Supplementary Figure S3**) and thereby has no impact on our objective to evaluate the distribution and unspecific clearance without TMDD. In order to describe TMDD, we extended the generic PBPK model by a reversible binding of the drug (D) to a target (R) to form a drug-target complex (C), target synthesis and turnover, and the degradation of the drug-target complex:

$$\frac{dD}{dt}=-k_{on}\cdot D\cdot R+k_{off}\cdot C$$

$$\frac{dC}{dt}=k_{on}\cdot D\cdot R-k_{off}\cdot C-k_{deg}\cdot C$$

$$\frac{dR}{dt}=k_{syn}\cdot \left(R_{0}-R\right)-k_{on}\cdot D\cdot R+k_{off}\cdot C$$

with the dissociation rate constant k_off_, the association rate constant k_on_ (k_on_ = k_off_/K_D_, using the equilibrium dissociation constant K_D_), the degradation rate constant k_deg_, the synthesis rate constant k_syn_, and the steady state target concentration R_0_.

### Model Development

The PBPK models for the antibodies were developed using PK data for adults. For palivizumab only the affinity to FcRn was fitted to match the experimental PK data for adults. We fitted the affinity to FcRn instead of using an experimentally determined value, Kd = 0.75 µM, (Suzuki et al., 2010), in order to avoid any potential bias introduced due to differences in the assay used to determine the Kd(FcRn) for palivizumab and the assays used for the compounds utilized in the development of the generic protein PBPK model (Niederalt et al., 2018). However, as shown below, the difference of the fitted value and experimentally determined was small. Since no experimental PK profiles for adults were found in the published literature, we adjusted Kd(FcRn) so that the simulated CL matches the CL reported in a population PK analysis for adults, 198 ml/day = 2.83 ml/day/kg (Robbie et al., 2012). For bevacizumab, the model was extended by processes representing TMDD in order to include also the reported sub-therapeutic dosages for adults (Gordon et al., 2001) for model building. The values from the PK-Sim expression database (Meyer et al., 2012) for VEGF-A for the relative expression across the different organs was used (expressed sequence tags, EST, from the UNIGENE database). The target was located in the extracellular space (plasma and interstitial space). The PBPK model was not extended by a tumor organ, i.e. VEGF-A was present only in the standard organs of PK-Sim. For the target affinity an experimental value was used (Papadopoulos et al., 2012) and for the target turnover rate, the value from a published compartmental TMDD model was used (Panoilia et al., 2015). The remaining parameters of the TMDD related extensions, i.e., the absolute target reference concentration (concentration in the organ with the highest tissue concentration) and the degradation rate constant of the drug-target complex, were fitted to the experimental concentration-time profiles for 0.1 to 10 mg/kg bevacizumab in adults together with the affinity to FcRn.

These parameters fitted to the adult PK data were kept constant for the pediatric PBPK models, i.e., PBPK simulations for children were predictions without adjusting model parameters to experimental PK data. The PBPK models developed for adults were scaled to children by adapting all anthropometric, anatomic, and physiological parameters *via* replacing the adult virtual individuals with the virtual pediatric individuals created from the PK-Sim databases and algorithms (Edginton et al., 2006; Willmann et al., 2007; Claassen et al., 2015).

Generally, the ICRP population (Valentin, 2002) was chosen for all adult and pediatric models from the PK-Sim database if not stated otherwise, i.e., for a given age, we generated a mean male virtual individual using the mean body height and weight from the ICRP population if not stated otherwise. The differences in PBPK simulation results between male and female individuals were small; cf. **Supplementary Figures S1A, B**. For the palivizumab model for premature infants and infants with bronchopulmonary dysplasia (Subramanian et al., 1998), also the “Preterm” population (Claassen et al., 2015) from the PK-Sim database was additionally tested (cf. **Supplementary Figures S2A, B**). In this study, the mean body weight reported for the population of chronically ill patients is considerably smaller than the mean body weight of the PK-Sim database for the reported age. Since the body height of the individuals was not reported and to avoid an unrealistic combination of body weight and height, we thus simulated two scenarios: First, we used the nominal age as reported for the study population together with the mean body weight from PK-Sim database. Second, we adjusted the age to match the body weight reported for the study population using the PK-Sim database. The anthropometric data of these scenarios are compared to the reported data in **Supplementary Table S1**.

Due to the long plasma half-lives of antibodies, the anatomy and physiology of young children changes during the plasma concertation measurements and the time-scale considered in the simulations even after a single dose application. Since these changes in anatomical and physiological properties might influence the PK on the time-scale considered in the simulations, two options were compared in the PBPK simulations: a) allowing no growth during aging, i.e., the physiological parameters stay constant during the simulation time, b) allowing growth during aging, i.e., the growth of the child during simulation was considered by dynamically updating the physiological parameters along the time scale of the simulation based on the human growth and maturation functions available from the PK-Sim database. Due to the increased volumes during growth, the plasma concentrations decrease with time leading to a higher apparent clearance in a NCA analysis of plasma concentration-time profile if growth is allowed.

### Model Parameters

#### Scaling Physiological Parameters to Children

The model was scaled to children by adjusting age-dependent anthropometric (body height and weight) and physiological parameters (blood flows, organ volumes, organ composition hematocrit, and cardiac output) according to the PK-Sim database (Edginton et al., 2006). While the knowledge on age-dependent changes of body composition regarding extracellular volume and blood plasma volume are implemented in the PK-Sim databases, there is currently no specific knowledge on the ontogeny of the additional antibody-specific distribution and clearance processes considered. Regarding these parameters the following assumptions were made (current default implementation in PK-Sim):

- Lymph flow: The lymph flow rates in PK-Sim are specified as fractions of the plasma flow rates for each organ. These fractions were assumed not to be age-dependent, i.e., the total absolute lymph flow scales as the plasma flow for each organ.
- Recirculation flow: The recirculation flow rates in PK-Sim are specified as fractions of the lymph flow rate scaling with an organ volume based allometric scaling factor of 2/3. This scaling exponent was used to scale between species during the development of the protein PBPK model in PK-Sim (Niederalt et al., 2018).
- The properties of vascular endothelium (endothelial pore radii, fraction of flow *via* large pores, and hydraulic conductivity of the vascular endothelium) were assumed to be constant throughout different ages.
- The concentration of free FcRn (combination FcRn and endogenous IgG), the specific endosomal uptake and recycling rate constants, and the specific endosomal clearance were assumed to be constant throughout different ages.
- In case of specific mechanisms pertinent to the monoclonal antibody, such as target-mediated clearance, the target concentration was assumed constant throughout different age groups.

#### Drug-Specific Parameters

The drug-specific properties used as input parameters for the PBPK model are given in **Table 2** together with the system-specific parameters related to the model extension describing TMDD.

**Table 2:** Input PBPK parameters used.

	Palivizumab	Bevacizumab
**Drug-specific input parameters**		
Molecular weight [kDa]	150^a)^	150^a)^
Hydrodynamic radius [nm]	5.34^b)^	5.34^b)^
Dissociation constant for FcRn binding [µM]	0.863^c)^	0.884^c)^
Equilibrium dissociation constant for target binding [nM]	NA	0.058^d)^
Dissociation rate constant for target binding [s^−1^]	NA	3.1E-5^d)^
**Physiological input parameters used for the model extension describing TMDD**		
Target concentration ^f)^ [µM]	NA	0.0113^c)^
Target synthesis rate constant [day^−1^]	NA	0.401^e)^
Degradation rate constant (drug-target complex) [day^−1^]	NA	0.0482^c)^

^a)^(Lobo et al., 2004); ^b)^(Taylor and Granger, 1984); ^c)^fitted to plasma concentration-time profiles for adults; ^d)^(Papadopoulos et al., 2012); ^e)^(Panoilia et al., 2015); ^f)^target concentration in the organ with the highest concentration (large intestine).

## Results

### Palivizumab

The Kd(FcRn) fitted to the adult palivizumab PK data had a value of 0.863 µM in good agreement with the experimentally measured value of 0.75 µM (Suzuki et al., 2010). The pediatric PBPK predictions for palivizumab were compared to the experimental data (Subramanian et al., 1998; Saez-Llorens et al., 2004) as well as the results derived from the population PK analysis by Robbie et al. (2012). **Figure 1** shows the comparison of observed and PBPK model predicted palivizumab plasma concentration-time profile for two different dosages in a pediatric population of infants with bronchopulmonary dysplasia or which are premature but without acute RSV infection (Subramanian et al., 1998). From **Figure 1**, it is evident that the PBPK model could reasonably well predict the observed data (the maximum deviation of predicted vs. observed concentrations is 54% for the 10 mg/kg dose and 39% for the 15 mg/kg dose over all prediction scenarios). Interestingly, the predictions not considering growth during the simulation time performed overall better compared to data from the chronically ill pediatric patients than the simulations including growth, at least for the dose of 15 mg/kg. For the 10 mg/kg and 15 mg/kg dose groups, this was also supported by comparing the model predicted PK parameters (area under the curve extrapolated to infinity, AUC_inf_, and terminal half-life [T_1/2_]) as described in **Table 3**. For both dosages, the model predicted PK parameters were within 20% difference compared to the reported parameters when growth was not included in the model. For the dose group of 3 mg/kg, the discrepancies between PBPK predictions and reported AUC values were higher (up to 60%) and the AUC was overestimated rather than underestimated as for the other dose groups. Correspondingly, for this dose group, the PBPK predictions of the reported PK parameters taking into account growth performed better than those not considering growth.

**Table 3:** Comparison of Model predicted and reported PK parameters of palivizumab.

Dose [mg/kg]	PK Parameters	Reported values from literature	Model Predicted: nominal age/with growth	Model Predicted: nominal age/no growth	Model Predicted: adjusted age/with growth	Model Predicted adjusted age/no growth
3^a)^	AUC_0-inf_ [µg/ml*days]	593	853 (1.44)^#^	943 (1.59)^#^	807 (1.36)^#^	923 (1.56)^#^
3^a)^	T_1/2_ [days]	19.3–26.8	19.4	22.1	19.6	23.2
10^a)^	AUC_0-inf_ [µg/ml*days]	3,508	2,843 (0.81)^#^	3,133 (0.89)^#^	2,694 (0.77)^#^	3,068 (0.87)^#^
10^a)^	T_1/2_ [days]	19.3–26.8	19.4	22.0	19.6	23.1
15^a)^	AUC_0-inf_ [µg/ml*days]	5,295	4,283 (0.81)^#^	4,697 (0.89)^#^	3,955 (0.75)^#^	4,554 (0.86)^#^
15^a)^	T_1/2_ [days]	19.3–26.8	19.4	21.8	19.6	23.4
5^b)^	AUC_0–30__days_ [µg/ml*days]	487	876 (1.80)^#^	925 (1.90)^#^	NA	NA
5^b)^	T_1/2_ [days]	22.6	19.6	23.5	NA	NA
15^b)^	AUC_0–30__days_ [µg/ml*days]	2386	2,791 (1.17)^#^	2,897 (1.21)^#^	NA	NA
15^b)^	T_1/2_ [days]	16.8	19.5	22.5	NA	NA

^a)^Reported values from literature (Subramanian et al., 1998); ^b)^Reported values from literature (Saez-Llorens et al., 2004); ^#^in brackets: ratio predicted/reported.

The discrepancies between the anthropometric data from the study population and the PK-Sim database (cf. section “Model Development”) were smaller if the PRETERM population was selected from the PK-Sim database using a gestational age of 26 or 30 weeks. However, the weight differences due to chronic illness seemed to be larger than the weight differences due to being born premature (cf. **Supplementary Table S1**). The predictions using the PRETERM population were similar to the predictions using the ICRP simulations (cf. **Supplementary Figures S2A–C**).

**Figure 2** shows the comparison of simulation results to experimental data for another clinical study (Saez-Llorens et al., 2004) where 15 mg/kg was given through intravenous route in pediatric patients with a median age of 5.2 months suffering from acute RSV infection. The clearance was underestimated by the simulations, and the higher apparent clearance of the simulation allowing growth seemed to capture the observed PK data better (the maximum deviation of predicted vs. observed concentrations is 27% for the simulation including growth and 39% without allowing growth). Comparing to the reported AUC, it was overestimated by around 20 % for the 15 mg/kg dose group (cf. **Table 3**). For the 5 mg/kg dose group, the predicted AUC was higher by 80 % if growth was taken into account and by 90 % if not (cf. **Table 3**).

### Bevacizumab

In **Figure 3**, observed plasma concentration-time profiles of bevacizumab in an adult population (Gordon et al., 2001) were compared to PBPK simulations for different dosages after intravenous application. After fitting the affinity to FcRn and the parameters related to target mediated clearance (cf. **Table 2**), the observed PK data were described reasonably well (the maximum deviation of predicted vs. observed concentrations is within 1.5 fold for the dosages 10, 3, and 1 mg/kg and within 2.1 fold for the lower dosages except for the first trough concentration of the 0.3 mg/kg dose for which the experimental mean concentration was reported to be higher than the concentration 7 days before).

Once the model was able to describe the PK of bevacizumab in the adult population, it was scaled to children assuming the same Kd(FcRn) and target related parameters as for the adult model. The predictions from the PBPK model were first compared to the mean experimental plasma concentration time profile of a 1- to 21-year-old population with a median age of 13 years (Glade Bender et al., 2008) in **Figure 4**. The age distribution of the trial patients was not reported. However, since the predictions of the published population PK model for the median age of 13 years are in good agreement with the experimental mean profile (cf. **Supplementary Figure S8**), using the median age of 13 years seems to be reasonable also for the PBPK predictions. We additionally added predictions for the minimum age of 1 year and for the adult for comparison as extremes of ages within the reported interval. **Figure 4** indicates that the predicted clearance was underestimated in comparison to the observed data. This is also apparent when comparing the PBPK predicted PK parameters to the reported PK parameters in **Table 4**. The AUC was overestimated up to 54% comparing the PBPK prediction for a 13 years old child. Like for the adult population, the target properties were consistently included in the model, however, as expected, for the pediatric dosages (5 and 15 mg/kg) target mediated clearance did not play a considerable role assuming the same target concentration and turnover as for the adult population (cf. **Supplementary Figure S3**).

**Table 4:** Comparison of PBPK model predicted and reported PK parameters of bevacizumab.

Dose [mg/kg]	PK Parameters	Reported values from literature^a)^	Predicted (PBPK) (13 years, with growth)
15	AUC_0-inf_ [µg/ml*days]	3180 ± 1655	4894
			(1.54)^#^
15	T_1/2_ [days] (2 weeks)	9.6 ± 4.4	19.7
			(2.05)^#^
15	V_ss_ [ml/kg]	66.2 ± 15	77.6
			(1.17)^#^
5	AUC_0-inf_ [µg/ml*days]	1115 ± 264	1,475
			(1.32)^#^
5	T_1/2_ [days] (2 weeks)	11.9 ± 1.7	18.5
			(1.55)^#^
5	V_ss_ [ml/kg]	73.7 ± 8.3	71.7
			(0.97)^#^

^a)^Mean values and standard deviation. Age range of patients included in the study was 1–21 years, median age, 13 years (Glade Bender et al., 2008); # in brackets: ratio predicted/reported.

**Figure 5** presents a comparison of weight adjusted clearance across different ages predicted by the PBPK model (10 mg/kg dose) with results from a published population PK analysis using an allometric scaling exponent of 0.75 for the clearance (Han et al., 2016b). The PBPK predictions show slightly lower weight adjusted clearances for younger children than for older in contrast to the estimates from the population PK analysis (Han et al., 2016b). Compared to the typical clearance estimated from the population PK analysis, the weight adjusted clearance was underestimated by around 10 % for adults and by 36 % for 1-year old children.

#### Comparison of Palivizumab and Bevacizumab

**Figure 6:**
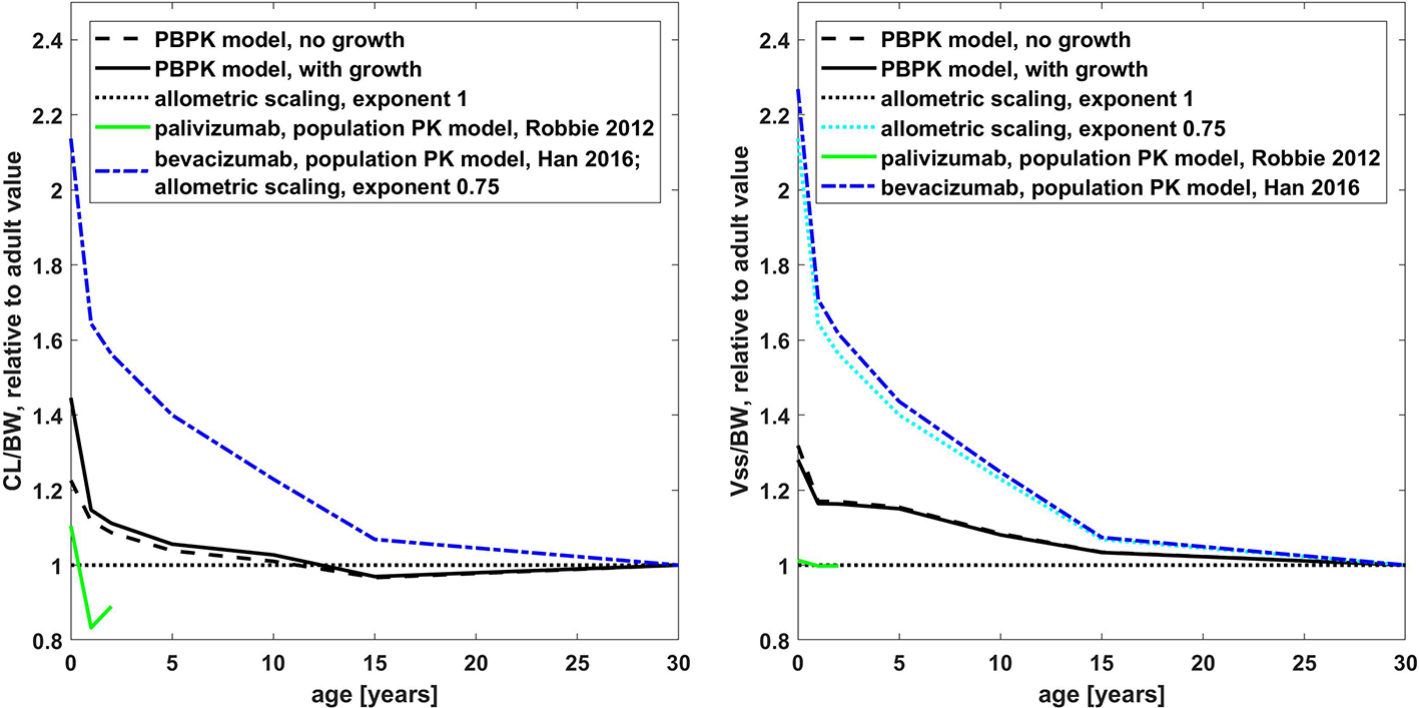
Comparison of the age dependency of the body weight normalized clearance (left hand side) and the steady state volume of distribution (right hand side) relative to the adult value for different simulations for palivizumab and bevacizumab (PBPK and published population PK models (Robbie et al., 2012; Han et al., 2016b)) and allometric scaling. The results of the published population PK models for palivizumab are shown up to an age of 2 years. Relative to the adult value, the PBPK simulations for palivizumab and bevacizumab are virtually the same (simulations for palivizumab are shown).

The age dependency of PK parameters simulated with the PBPK model is compared to the simulations with the published population PK models for palivizumab and bevacizumab and to allometric scaling in **Figure 6**. Relative to the adult value, the PBPK simulations for palivizumab and bevacizumab were virtually the same (below 2% difference for the PK parameters shown in **Figure 6**; cf. also **Supplementary Figure S4** for a direct comparison of palivizumab and bevacizumab PBPK simulations for different age groups). From **Figure 6**, it can be seen that the PK of palivizumab and bevacizumab simulated by the published population PK models was different, and the PBPK predictions lie in between the predictions from the population PK models for palivizumab and bevacizumab.

The population PK model for bevacizumab used an allometric scaling exponent of 0.75 leading to an increase in body weight normalized clearance by a factor of 2.14 for newborns relative to the adult value, which corresponds to a reduction of exposure to 47% for newborns.

In the published population PK model for palivizumab, the clearance was described by allometric scaling with an exponent of 0.75 in combination with a maturation function (Robbie et al., 2012). The clearance maturation function compensates the allometric scaling with an exponent of 0.75 so that the clearance combining allometric scaling and the maturation function was effectively similar to an allometric scaling with an exponent near to 1. Additionally, growth was taken into account for this model which increases the apparent clearance in the NCA analysis. Together, this led to a decrease in body weight normalized clearance by a factor of 0.83 (120% increase in exposure) for 1-year old children and an increase by a factor of 1.1 (decrease to 91% in exposure) for newborns relative to the adult value. The behavior of the PBPK model was in between the two population PK models showing a clearance scaling which is similar to an allometric scaling with an exponent of 0.928 if growth during simulation was not taken into account. With growth, the PBPK model performed similar to an allometric scaling with an exponent of 0.896 (cf. **Supplementary Figure S6**). Considering growth in the PBPK model led to an increase in the apparent clearance for young children, of around 20% for newborns compared to the simulation without taking growth into account.

Also, with respect to the scaling of the volume of distribution, the behavior of the PBPK model was in between the behavior of the published population PK models for palivizumab and bevacizumab. While the central as well as the peripheral compartment volumes were scaled with an exponent of 1 for the population PK model of palivizumab, the volumes were scaled with an exponent of 0.701 and 0.766, respectively, for the population PK model of bevacizumab.

## Discussion

PBPK modeling and simulation approaches have gained popularity in recent years, particularly for predicting the impact of drug-drug interactions, selecting an optimal dose and clinical trial design for pediatric applications, and for characterizing the impact of organ impairment (Zhao et al., 2012; Jiang et al., 2013; Lagishetty et al., 2016). PBPK models typically consist of three distinct parts: 1) drug-specific parameters characterizing the physicochemical properties of the drug, 2) system-specific parameters characterizing the functioning of the underlying system, and 3) trial design parameters. Since PBPK models are an ideal platform for evaluating the combined impact of drug- and system-specific factors on the PK of drugs, it can be used as a means of identifying an effective dose in pediatric populations after incorporating the age-related changes in the system information without modifying the drug properties. Several groups have reported promising results in using PBPK modeling to predict pediatric drug concentrations for all age groups for a variety of small molecule drugs (Edginton et al., 2006; Johnson et al., 2006; Parrott et al., 2011; Jiang et al., 2013; Maharaj et al., 2013). While these PBPK models have been increasingly used to predict drug PK in children for small molecules, the applicability for the increasingly important drug class of large molecules such as biologicals, including antibodies, has received little attention so far.

Some of the knowledge implemented in PBPK models for small molecules is also relevant for therapeutic proteins, e.g., the age-dependent change of body composition regarding extracellular volume and blood plasma volume. However, for many mechanisms influencing the PK of therapeutic proteins, there is only limited quantitative information regarding their maturation (Xu et al., 2013; Edlund et al., 2015; Liu et al., 2019).

The current knowledge on pediatric physiology and maturation processes has been recently reviewed with a special focus on mechanism relevant for PBPK models of antibodies (Malik and Edginton, 2018). In this review, the median of FcRn expression in rats over several organs was plotted against age showing a tendency for lower FcRn expression in very young rats. The data for the single organs showed also high fluctuations during aging (Tian et al., 2014). Together with the relatively high concentrations of endogenous IgG after birth, the lower FcRn concentration would lead to a higher body weight-normalized clearance of therapeutic proteins for very young children aside from other mechanisms (Malik and Edginton, 2018). It was further hypothesized, that there is a faster extravasation in young children due to a relatively higher ratio of tissues with leaky vascular endothelium and higher capillary density in young children (Malik and Edginton, 2018).

However, there is still a high degree of uncertainty associated with an *a-priori* parametrization for humans derived from the currently available physiological data. Our aim was thus to assess in a first step PBPK predictions incorporating the existing knowledge regarding physiological changes relevant for small molecules, but assuming no age-dependent changes regarding the additional antibody-specific parameters like FcRn concentration, endogenous IgG concentration, and properties of the vascular endothelium. The lymph and recirculation flow rates were changed with the organ volumes in the same way as for scaling between species in the PK-Sim PBPK model (Niederalt et al., 2018). This assessment of PBPK predictions can then serve as a basis to reveal knowledge gaps in the ontogeny of relevant physiological parameters and available PK information.

For palivizumab, the PBPK predictions did overall reasonably well describe the experimental data. The comparison of the PBPK predictions with observed concentration-time profiles for children with chronic lung disease and/or premature children (Subramanian et al., 1998) showed a slight underestimation of AUC (or overestimation of apparent clearance) for the 10- and 15-mg/kg dose groups, especially when growth was considered in the simulation. A possible reason for the slightly worse predictions when growth was considered is that the normal growth rate in the PK-Sim database is overestimating the growth rate of chronically ill pediatric patients. In line with that assumption, the body weights of the children reported in the study were considerably lower than the mean body weights in the PK-Sim database for that age (cf. **Supplementary Table S1**). The PBPK predicted clearance for palivizumab was also higher (up to a factor of 1.5) when compared to the clearance of the mean population PK model (Robbie et al., 2012). In contrast, when the PBPK predictions were compared to the concentration-time profile from children with acute RSV infection or with the PK parameters from the 3-mg/kg dose group of the study with children with chronic lung disease and/or premature children, the apparent clearance was underestimated even when growth was considered in the simulation. In summary, no common trend was identified for palivizumab that allowed for consistent quantification of missing ontogeny for a generally relevant process or parameter like the free FcRn concentrations. The reasons for the slight differences in the PK observed in the palivizumab studies with RSV infected children vs. children with chronic lung disease/premature children or within different dose groups are unclear. We cannot rule out that an essentially increased elimination in children due to ontogeny was partly masked by disease effects.

For bevacizumab, the PBPK prediction for children underestimates the clearance compared to the mean plasma concentration time profile merging the age range from 1 year to 21 years with a median age of 13 years (Glade Bender et al., 2008), cf. **Figure 4**, as well as compared to the results from the published population PK model (Han et al., 2016b), cf. **Figure 5**.

The change of PK with age of palivizumab and bevacizumab was considerably different, at least according to the published population PK models. While for palivizumab the clearance was described by allometric scaling with exponent 0.75 in combination with a maturation function (Robbie et al., 2012), it was described by a pure allometric scaling with exponent 0.75 for bevacizumab (Han et al., 2016b). Interestingly, the total clearance combining allometric scaling and the maturation function for the published palivizumab model was effectively similar to an allometric scaling with an exponent near to 1 (cf. **Figure 6**). Note that growth during simulation time was not considered in the population PK model of bevacizumab which seems to be more than compensated for by the smaller exponent. Also, with respect to the volume of distribution, the population PK models for palivizumab and bevacizumab are different. An exponent of 1 was used for the palivizumab model for the allometric scaling of the central and peripheral volume, even though the proportion of extracellular water is higher in children compared to adults (Edginton et al., 2006). For the bevacizumab model an exponent < 1 was used to describe the data (using an allometric scaling exponent of 1, leads to overestimation of the plasma concentrations, cf. **Supplementary Figure S8**).

The reason for the differences in the PK of palivizumab and bevacizumab according to the population models is not clear. Both antibodies are humanized IgG1 antibodies, and there is no indication of considerable target effects at usual therapeutic dosages (cf. also the small target effect in the pediatric PBPK simulations for bevacizumab shown in **Supplementary Figure S3**). Partly these differences might not reflect real PK differences but rather limitations of the different modeling approaches (e.g. regarding scaling of clearance and volumes) chosen in the population PK analyses which naturally already represent an interpretation of sparse data. Thus, for most individuals included in the population PK analysis of palivizumab only trough concentrations were measured. Since there are no goodness of fit plots for different age groups published for both population PK analyses, and there are neither concentration time profiles nor PK parameters published for different age groups for bevacizumab, it is not clear if the accuracy of the published population PK models of both compounds is age-dependent.

Other possible reasons for the differences found in the population PK analyses of palivizumab and bevacizumab are differences in the studied populations, especially in the disease state. Thus, it was found that the bevacizumab clearance is lower in CNS tumor patients compared to sarcoma patients (Han et al., 2016b). The reason for this reduced clearance is not clear. It is known, that general proteolytic degradation and thereby antibody clearance can be affected by disease states including cancer, inflammatory conditions, and cachexia. Accordingly, serum albumin levels are often used as covariate for antibody clearance (Ryman and Meibohm, 2017). Since albumin levels have been included also as a covariate in the population PK analysis of bevacizumab (Han et al., 2016b), Han et al. discuss further hypotheses on the underlying mechanisms responsible for the PK differences in CNS tumor patients and sarcoma patients including different levels of anti-drug antibodies due to a different degree of tumor-induced inflammation, tumor burden, and concomitant medications (Han et al., 2016b). The clearance of bevacizumab in patients with CNS malignancies under the age of three years was reported to be even lower than estimated from the clearance of the population PK model published by Han et al. in two of three cases (Gojo et al., 2017). Any disease-specific changes in physiology were not considered in the PBPK model.

With respect to the change of clearance as well as the change of the volume of distribution with age, the behavior of the PBPK model was in between the behaviors of the published population PK models of palivizumab and bevacizumab. The generic PBPK model (without additional mechanisms like TMDD being effective) shows a slight increase of body weight-normalized clearance with the current approach assuming an age-independent FcRn concentration and endogenous IgG concentration. The behavior of the PBPK model is similar to an allometric scaling with an exponent of 0.928 if growth during simulation is not taken into account. The reason for this is the age-dependent change in vascular volume and—proportional to that—the endosomal volume per body weight (cf. **Supplementary Figure S5**). With the same approach as used in the present study, it was recently shown that this leads accordingly to a slightly higher body weight-normalized dose in younger children compared to adults in order to obtain equivalent exposure (Hanke et al., 2019).

The behavior of the PBPK model with respect to the volume of distribution is governed by the age-dependent changes in the body composition leading to an increase of extracellular space for younger children (Edginton et al., 2006), as well as the scaling of the lymph and fluid recirculation flow rates.

The simulation results shown in the present study show that for children younger than 6 months, the increase in volumes of body compartments due to growth might in certain cases be relevant for the plasma-concentration time profiles due to the long half-life of antibodies. For newborns, the apparent clearance due to this growth was higher by around 20% compared to simulations for which growth was not taken into account.

The same published population PK models as used in the present study, were also recently used in a minimal PBPK study addressing the developmental PK of antibodies (Hardiansyah and Ng, 2018). In that study, the FcRn concentration, the synthesis rate of endogenous IgG, and the extravasation rate were fitted to the simulations results of the population PK model of bevacizumab and to endogenous IgG levels for different age groups. The final minimal PBPK model was then evaluated using the simulation results of the population PK model of palivizumab. The minimal PBPK model was able to reasonably well predict the palivizumab PK represented by simulations using the originally published maturation function. However, using the revised maturation function referenced in the erratum of the palivizumab population PK analysis (Robbie et al., 2012), the clearance of palivizumab in young children is considerably smaller than for bevacizumab based on the published population PK models as discussed above. Thus, in order to identify the ontogeny of FcRn based on PK data, further research is needed in order to understand the underlying mechanisms which lead to the differences in the population PK descriptions of the two antibodies.

While Hardiansyah and Ng added ontogenies to the concentrations of FcRn and endogenous IgG as described above, Malik and Edginton (2020) have chosen different parameters in a recent publication to introduce ontogeny to clearance and additionally introduced ontogeny to parameters influencing the volume of distribution. Using also PK-Sim, they introduced ontogenies for the capillary density, for lymph flow, and for leukocyte concentration in addition to the scaling rationale used in the present study. The ontogeny for the capillary density and the leukocyte concertation resulted in an additional ontogeny for the endosomal volume influencing the clearance. With these ontogenies they were able to predict the PK of the antibodies pagibaximab, palivizumab, MEDI 8897 and IVIG. Interestingly, the predictions for palivizumab were in good agreement with the observed data introducing an ontogeny leading to a higher clearance compared to our simulations. However, they used an experimentally determined affinity to FcRn, K_D_ = 0.75 µM (Suzuki et al., 2010), without confirmation that this value is consistent with the adult PK of palivizumab. This value, which is slightly lower than the value used in the current study (fitted to the observed clearance in adults), could have partly compensated for the increase in clearance due to the ontogeny introduced.

In the present study, we focused on a PBPK model representing generic mechanism and further drug-specific mechanisms like TMDD were considered not to be relevant at therapeutic dosages for the examples discussed. PBPK models can readily be extended in order to take into account TMDD related processes as described above. However, drugs for which TMDD considerably influences the PK, the ontogeny of TMDD related parameters have to be known in order to scale the PBPK model from adults to children. Especially the target concentration is considered to potentially depend on the age but also on the disease state (Malik and Edginton, 2018). PBPK predictions, taking TMDD and ADAs into account, were recently compared to allometric scaling for the example of infliximab (Malik and Edginton, 2019). It was found that for children older than 4 years, both methods showed comparable accuracy. PBPK modeling was more accurate for predicting volume of distribution and for predicting PK for exposure scenarios which were not used to calibrate the allometrically scaled models. On the other hand, the allometrically scaled models were more accurate for predicting clearance (Malik and Edginton, 2019) without considering additional ontogeny on e.g. TMDD.

In summary, the predictions for the two case examples yielded no consistent results which would allow a quantification of missing ontogeny of antibody PK-specific mechanisms in the current approach. However, often an increase in body weight normalized clearance of therapeutic proteins is observed in young children compared to adults (Shi and Derendorf, 2010; Edlund et al., 2015; Zhang et al., 2015) and Malik and Edginton (2020) obtained recently good PBPK predictions with adding additional ontogeny. Thus, additional ontogeny for elimination relevant processes should be considered for PBPK modeling supported dosing selection for pediatric trials. Together with this recent work by Malik and Edginton (2020), the present work provides an important contribution to the physiological understanding of antibody PK in children but also identifies knowledge gaps due to the lack of experimental data.

## Data Availability Statement

All datasets generated for this study are included in the article/**Supplementary Material**.

## Author Contributions

SB, CN, TE, J-FS, and SS contributed to the study conception and design. SB, SS, and CN contributed to PBPK model development and simulations. YL and VV performed the allometric scaling simulations. CN, SB, YL, and SS drafted the manuscript. All authors contributed to the article and approved the submitted version.

## Funding

This work was supported by Bayer Technology Services GmbH, now Bayer AG.

## Conflict of Interest

This work was supported by Bayer Technology Services GmbH, now Bayer AG. CN, J-FS and TE are employed by Bayer AG and TE is a Bayer AG share holder. SB, YL, and SS received funding by Bayer Technology Services GmbH, now Bayer AG.

The remaining author declares that the research was conducted in the absence of any commercial or financial relationships that could be construed as a potential conflict of interest.
