# Supplementary material for: Physiologically Based Pharmacokinetic Modeling of Monoclonal Antibodies in Pediatric Populations Using PK-Sim
Source: Front Pharmacol. 2020 Jun 11;11:868. doi: 10.3389/fphar.2020.00868 (PMC7300301; doi:10.3389/fphar.2020.00868)
Supplement: Supplementary file 1 [file DataSheet_1.pdf]

Physiologically-based Pharmacokinetic (PBPK) Modeling of Monoclonal Antibodies in Pediatric Populations Using PK-Sim

Supplementary Information

Sumit Basu, Yi Ting (Kayla) Lien, Valvanera Vozmediano, Jan Schlender, Thomas Eissing, Stephan Schmidt, Christoph Niederalt

Contents

1 Comparison of PBPK results for female vs. male pediatric individuals for palivizumab 2

2 PBPK prediction for palivizumab using PRETERM individuals and ICRP individuals 3

3 Effect of target mediated drug disposition on the PBPK results for pediatric simulations for bevacizumab 6

4 Comparison of simulated PBPK and 2-compartmental model profiles for palivizumab and bevacizumab 7

5 Scaling behavior of the clearance resulting from the PBPK model 8

6 Allometric Scaling 9

6.1 Methods 9

6.2 Results 9

7 References 13

1 Comparison of PBPK results for female vs. male pediatric individuals for palivizumab

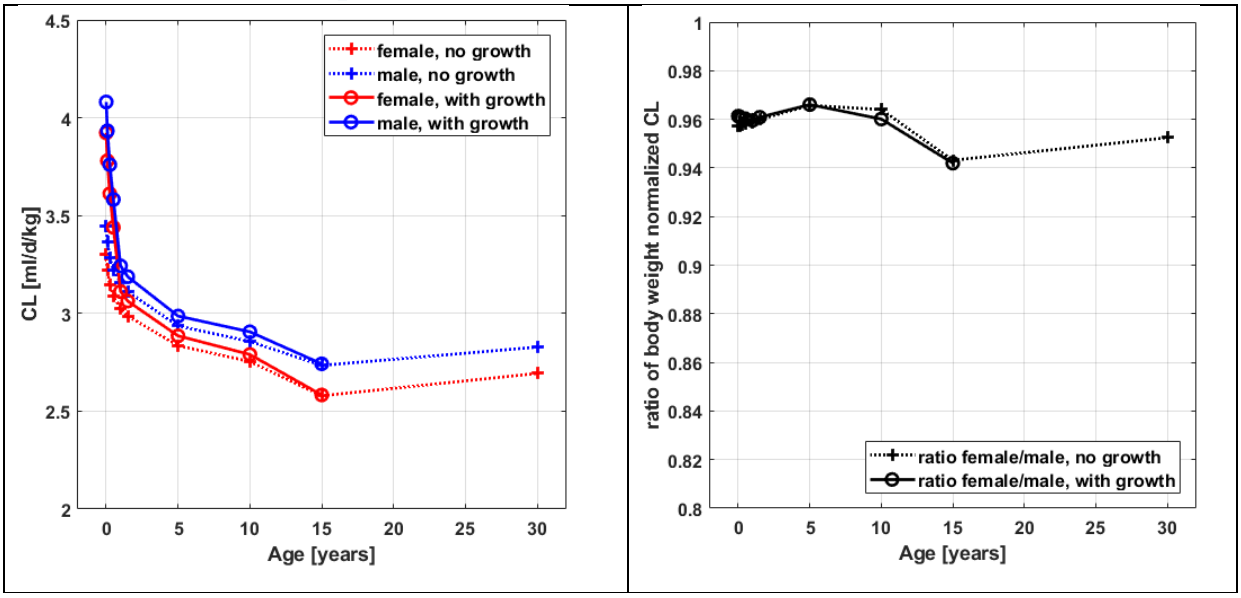

Figure S1a: Comparison of body weight normalized clearance from PBPK simulations for a mean female and mean male individual for different ages (ICRP population) using the palivizumab model as example. Simulations taking into account growth during the simulation time are shown as well as simulations with constant body weight during simulation time (no growth). Left hand side: body weight normalized clearance vs. age for female and male individuals, right hand side: ratio of body weight normalized clearance female/male.

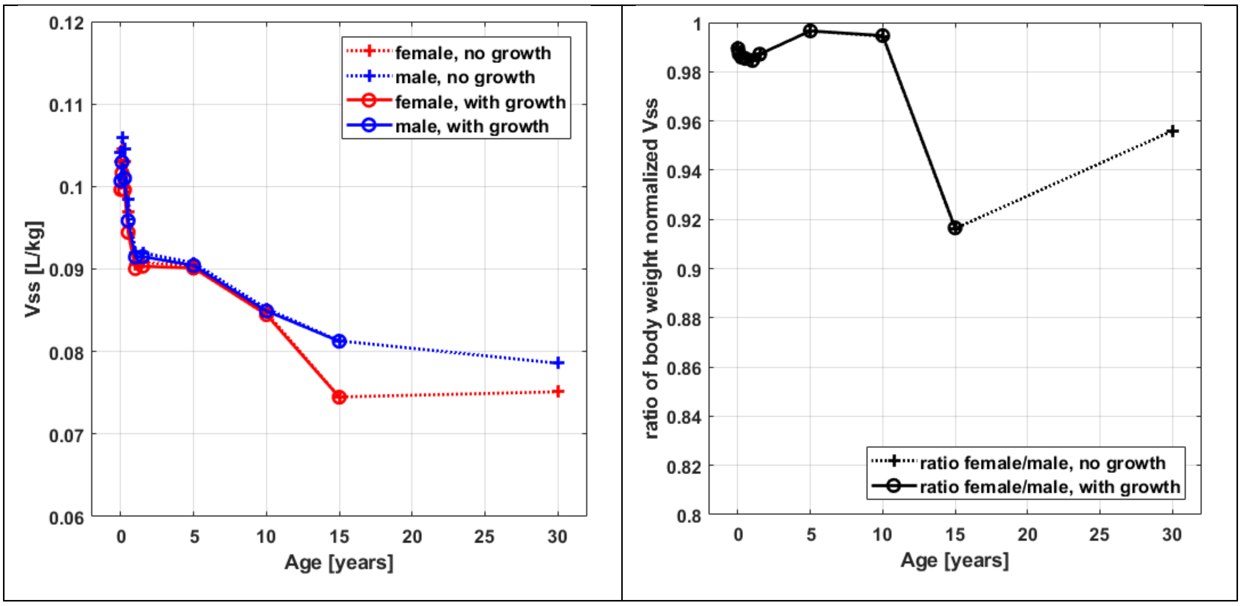

Figure S1b: Comparison of body weight normalized steady state volume of distribution from PBPK simulations for a mean female and mean male individual for different ages (ICRP population) using the palivizumab model as example. Simulations taking into account growth during the simulation time are shown as well as simulations with constant body weight during simulation time (no growth). Left hand side: body weight normalized steady state volume of distribution vs. age for female and male individuals, right hand side: ratio of body weight normalized steady state volume of distribution female/male.

2 PBPK prediction for palivizumab using PRETERM individuals and ICRP individuals

For the palivizumab model for premature infants and infants with bronchopulmonary dysplasia (Subramanian et al., 1998), also the “Preterm” population (Claassen et al., 2015) from the PK-Sim database was additionally tested for the comparison with the plasma concentration-time profiles of the 10 mg/kg and 15 mg/kg dose groups. For the mean individual for this study, a gestational age of 26 or 30 weeks was assumed taking into account that 80 and 90.9% of the children had a gestational age ≤ 32 weeks in the 10 mg/kg and 15 mg/kg dose groups, respectively.

Two scenarios were used: First, the nominal age as reported for the study population was used together with the mean body weight from PK-Sim database. Second, we adjusted the age to match the body weight reported for the study population using the PK-Sim database.

Table S1: Comparison of anthropometric data from individuals of the study of palivizumab in children which had bronchopulmonary dysplasia or are born premature (Subramanian et al., 1998) with anthropometric data from the PK-Sim database using the Preterm (Claassen et al., 2015) or the ICRP (Valentin, 2002) population.

Population	mean gestational age [weeks]	mean age [months]	mean weight [kg]
15 mg/kg group from palivizumab study (Subramanian et al., 1998)	90.9% of individuals ≤ 32	8.1+/-1.7	4.9
Preterm, nominal age	26	8.1	6.85
Preterm, adjusted age	26	5.7	4.9
Preterm, nominal age	30	8.1	7.2
Preterm, adjusted age	30	4.9	4.9
ICRP, nominal age	N.A.	8.1	7.9
ICRP adjusted age	N.A.	2.6	4.9
10 mg/kg group from palivizumab study (Subramanian et al., 1998)	80% of individuals ≤ 32	7.3+/-2	5.4
Preterm, nominal age	26	7.3	6.2
Preterm, adjusted age	26	6.3	5.4
Preterm, nominal age	30	7.3	6.6
Preterm, adjusted age	30	5.7	5.4
ICRP, nominal age	N.A.	7.3	7.5
ICRP adjusted age	N.A.	3.5	5.4
3 mg/kg group from palivizumab study (Subramanian et al., 1998)	100% of individuals ≤ 32	6.9+/-1.3	5.2
ICRP, nominal age	N.A.	6.9	7.2
ICRP adjusted age	N.A.	3.2	5.2

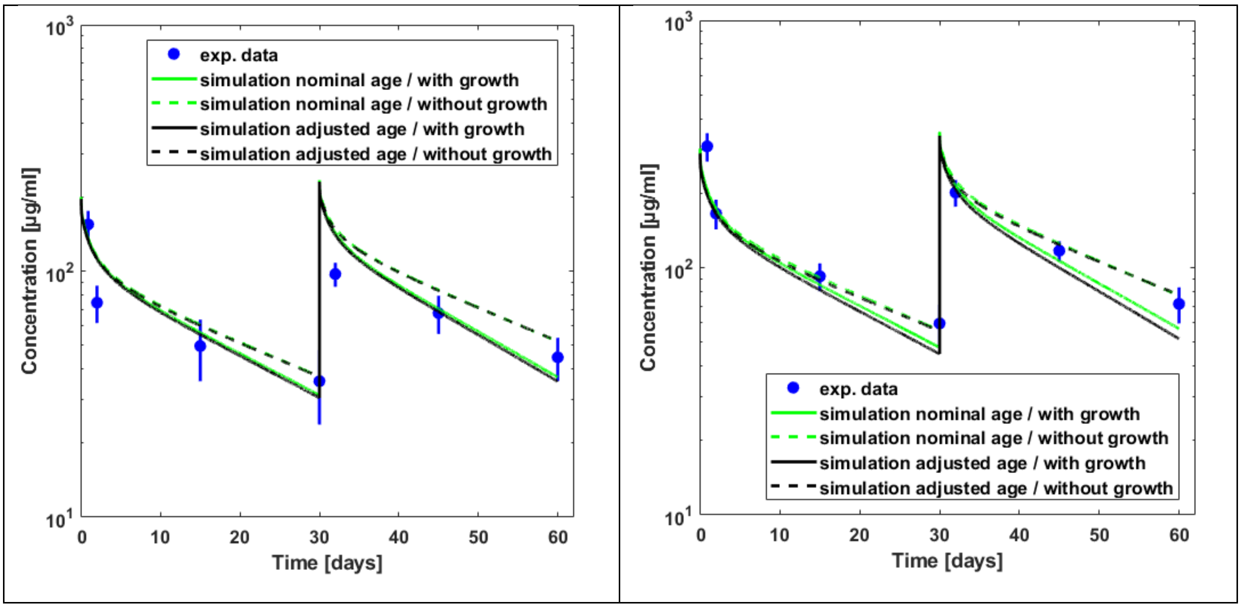

Figure S2a: Comparison of predicted PK using PRETERM individuals with a gestational age of 26 weeks with experimental data (Subramanian et al., 1998) for 10 mg/kg (left hand side) and 15 mg/kg (right hand side).

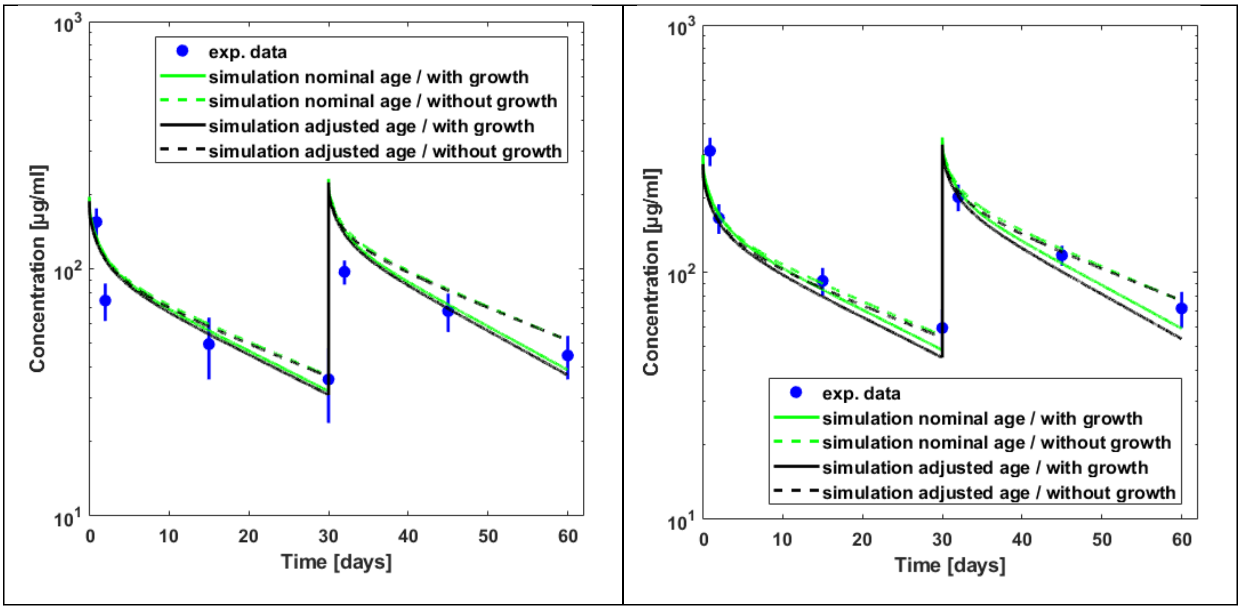

Figure S2b: Comparison of predicted PK using PRETERM individuals with a gestational age of 30 weeks with experimental data (Subramanian et al., 1998) for 10 mg/kg (left hand side) and 15 mg/kg (right hand side).

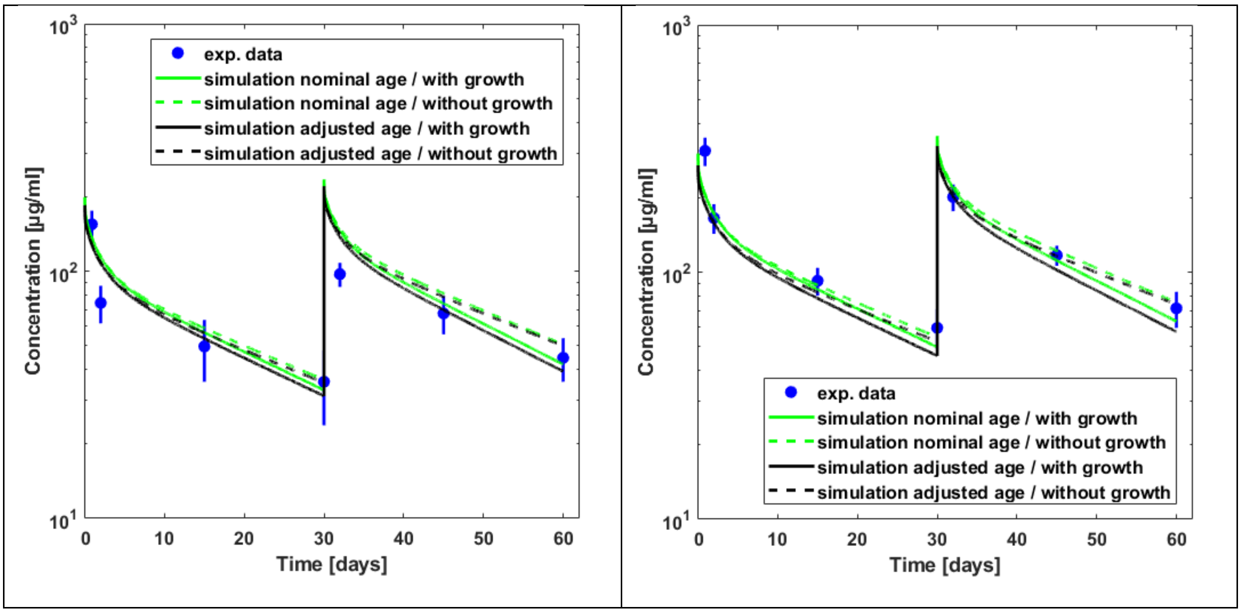

Figure S2c: Comparison of predicted PK using ICRP individuals with experimental data (Subramanian et al., 1998) for 10 mg/kg (left hand side) and 15 mg/kg (right hand side).

3 Effect of target mediated drug disposition on the PBPK results for pediatric simulations for bevacizumab

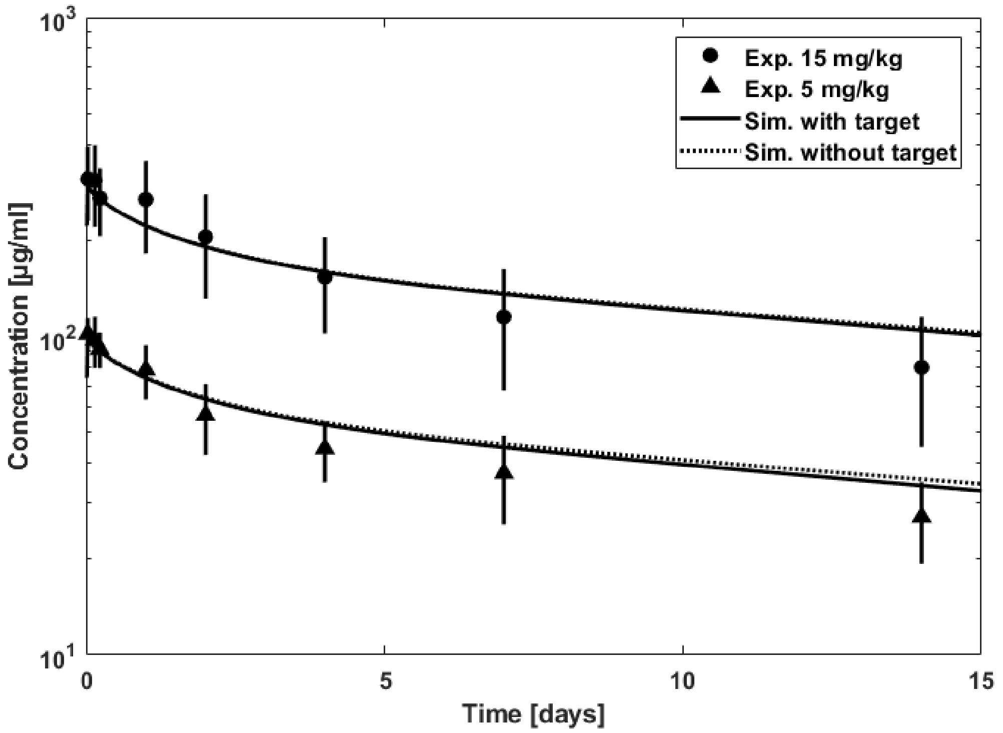

Figure S3: Comparison of PBPK simulations taking target into account vs. simulations taking target not into account (setting target concentration to 0) for bevacizumab for a virtual child of age 13 years (with growth). Experimental data are taken from literature (Glade Bender et al., 2008).

4 Comparison of simulated PBPK and 2-compartmental model profiles for palivizumab and bevacizumab

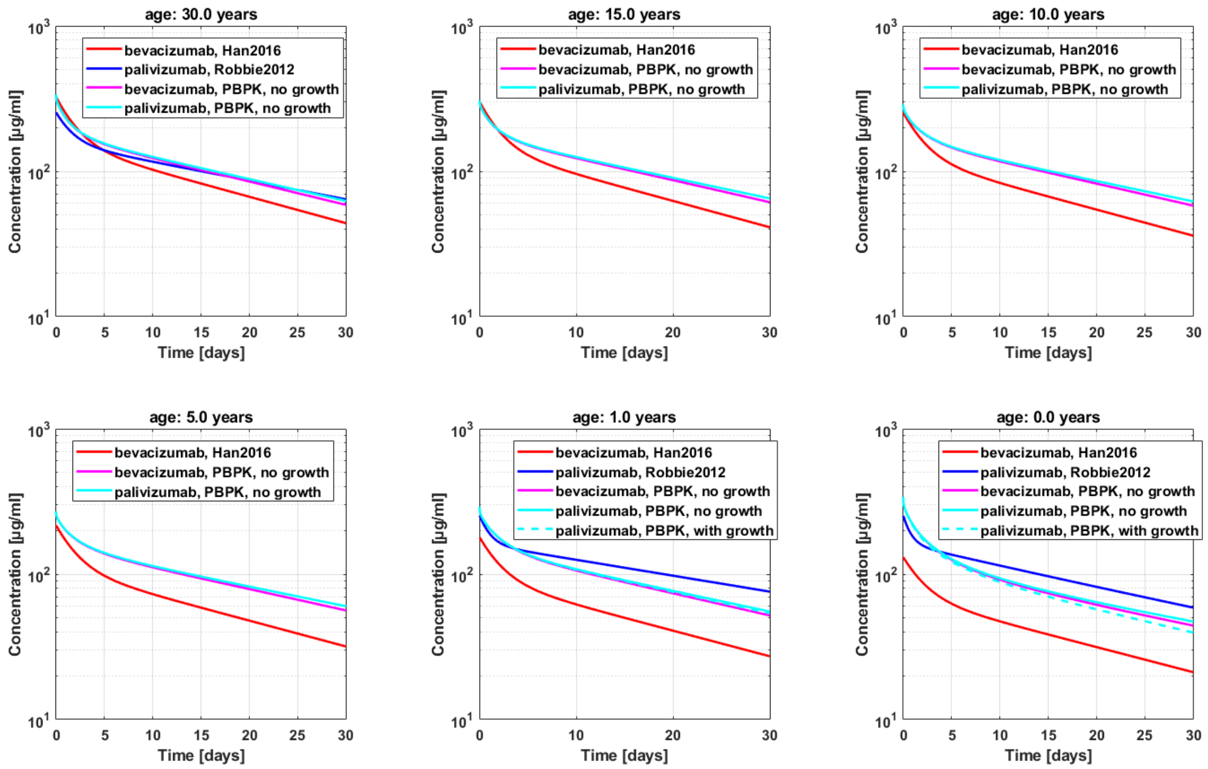

Figure S4: Comparison of different model simulations across different ages for palivizumab and bevacizumab (Dose: 15 mg/kg). The red and blue lines refer to the published 2-compartmental models based on the population PK analyses for bevacizumab (Han et al., 2016) (typical male sarcoma patient) and palivizumab (Robbie et al., 2012) (typical patient without further covariates and anti-drug-antibody titer).

5 Scaling behavior of the clearance resulting from the PBPK model

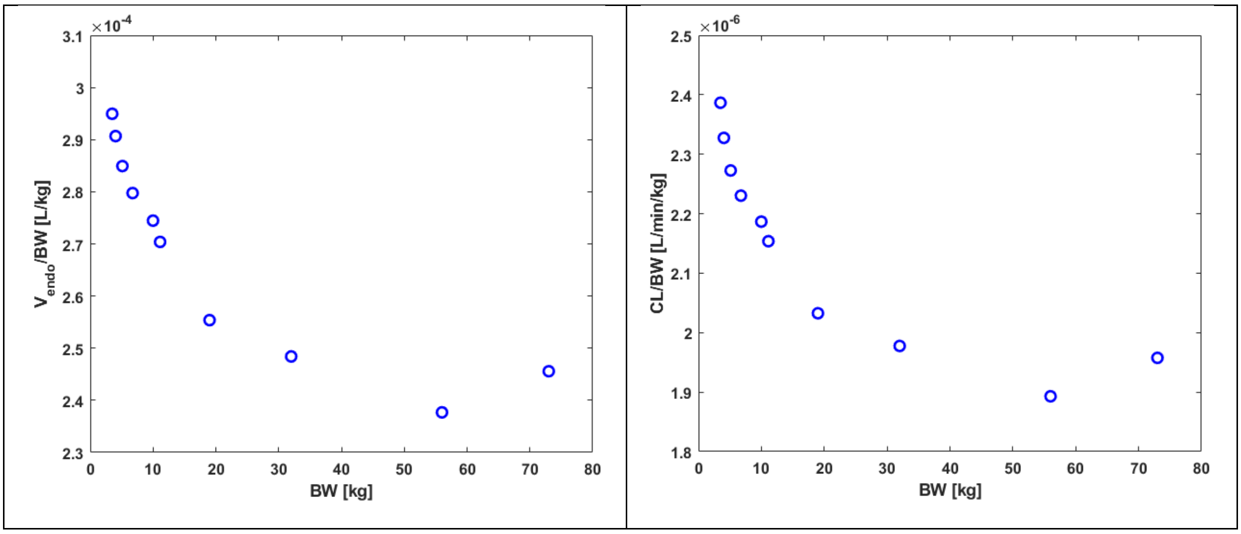

Figure S5: Endosomal volume (sum over all organs) per body weight over body weight (left hand side) and body weight normalized clearance of palivizumab PBPK model without aging over body weight (right hand side).

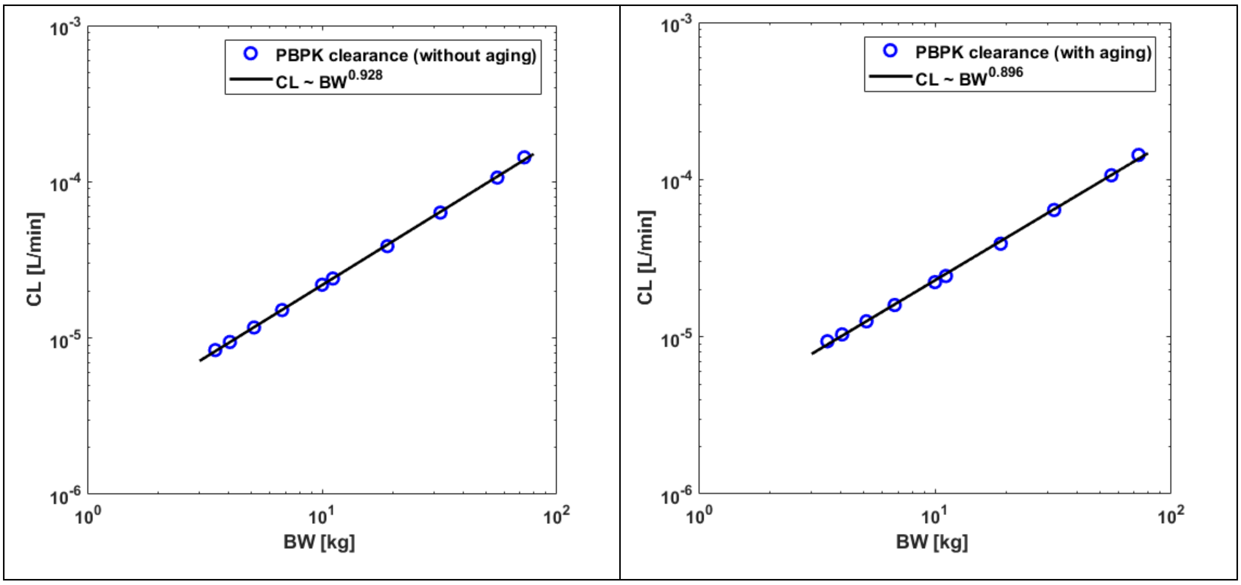

Figure S6: Symbols: Clearance of palivizumab PBPK model without aging (left hand side) and with aging (right hand side) over body weight. Black line: fitted regression line, equation CL = 2.57E-6 * $BW^{0.928}$ for the clearance without aging and CL = 2.91E-6 * $BW^{0.896}$ for the clearance with aging.

6 Allometric Scaling

6.1 Methods

To compare the performance of the PBPK model, a simple allometric approach was applied to assess the exposure in the pediatric population ranging from 0-20 years old for bevacizumab. A 2-compartment model was fitted to the published adult data (Gordon et al., 2001) using the highest doses of 10, 3 and 1 mg/kg. The adult 2-compartment model was scaled to children using an allometric exponent of 0.75 for clearance as well as intercompartmental-clearance and an exponent of 1 for the central and peripheral volumes of distribution. The allometric scaling approach was performed using the modeling software tools MoBi (version 8.0) and Phoenix WinNonlin 64 (version 8.1).

6.2 Results

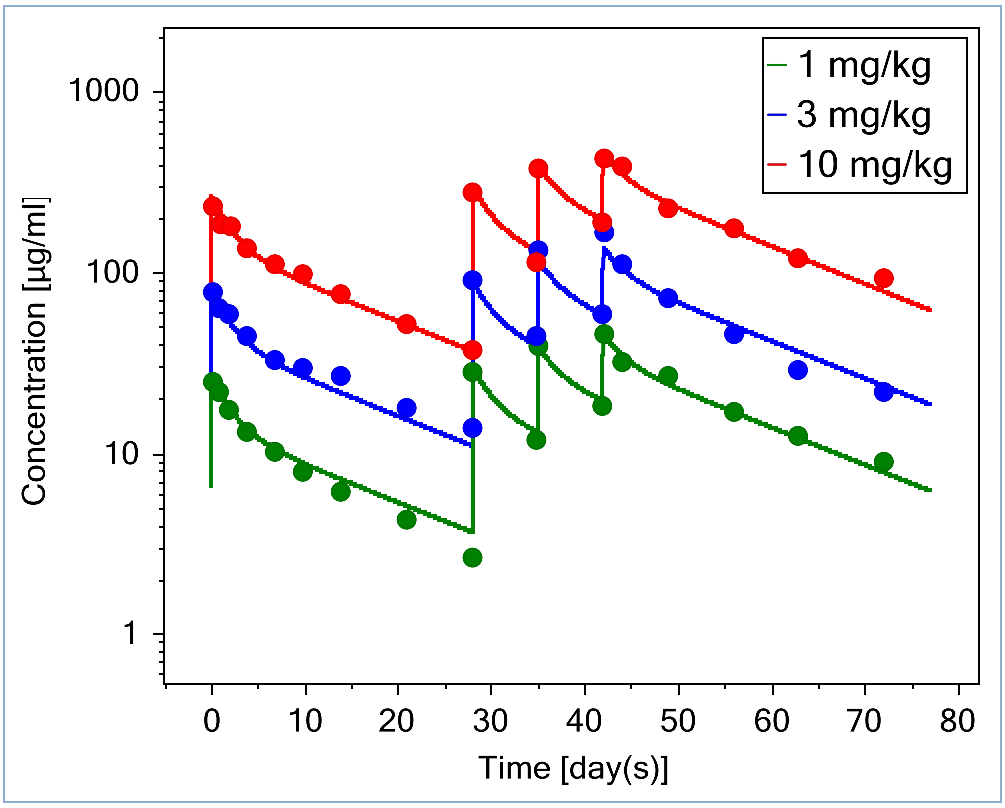

Figure S7: Comparison of plasma concentration-time profiles of bevacizumab simulated with the 2-compartment model (lines) to experimental profiles for adults used for parameter identification (Gordon et al., 2001).

Table S2: Parameters of the 2-compartment model for adults (body weight 70 kg) fitted to the plasma concentration-time profiles for the 10, 3 and 1 mg/kg dosages (Gordon et al., 2001).

$Volume_{central}$ [ml]	$Volume_{peripheral}$ [ml]	Intercompartmental-clearance [ml/h]	Clearance [ml/h]
2649	1612	17.8	9.18

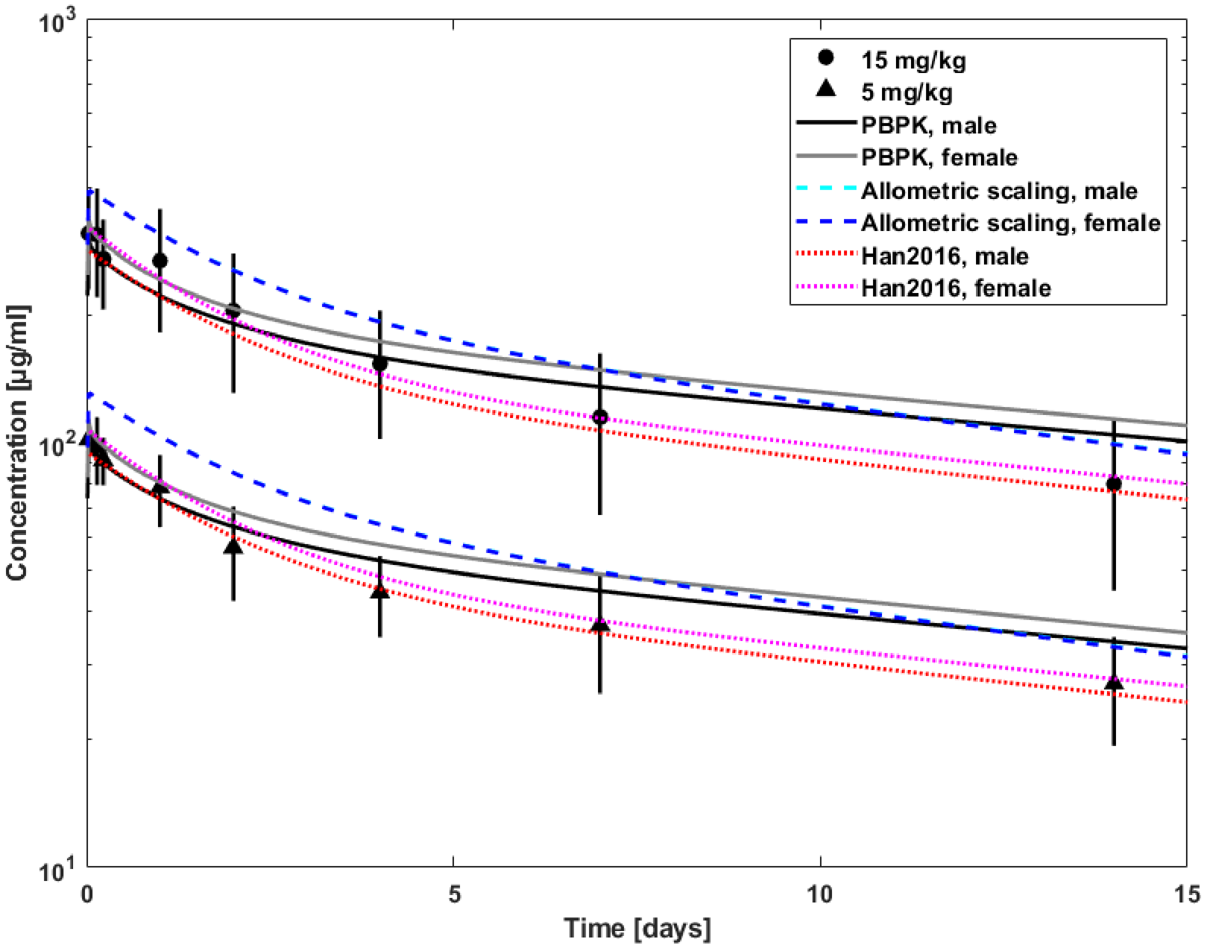

Figure S8: Comparison of PBPK simulations (with growth), simulations from the allometrically scaled 2-compartmental model and simulations from 2-compartment model from the published population PK analysis (Han et al., 2016) to experimental data from pediatric patients (Glade Bender et al., 2008). The experimental profiles represent mean concentrations from a population with an age range from 1-21 years, median age 13 years. For the simulations, an age of 13 years was assumed using the corresponding body weights for a male and female individual (ICRP). The allometric scaling results for the male and female individual are virtually the same (profiles lie on top of each other). For the simulations of the published population PK model, a sarcoma tumor type was assumed.

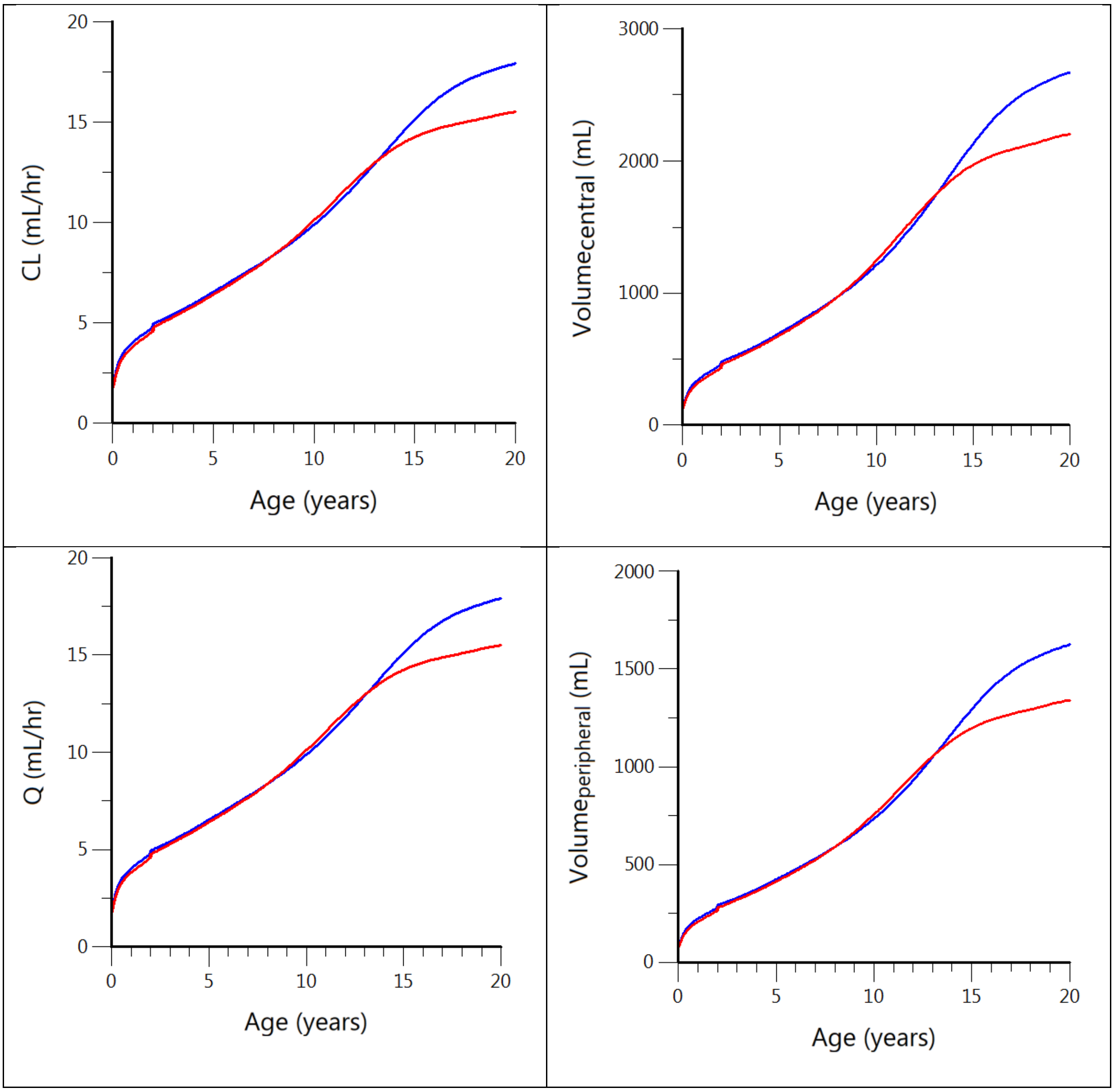

Figure S9: Allometric scaled 2-compartment model of bevacizumab. Body weights were obtained from the growth charts published from World Organization Growth standard tables (0-2 years old) and Center for Disease Control standard tables (3-20 years)

Top left hand side: Comparison of clearance versus different ages simulated by allometric scaling model of bevacizumab for male (blue line) and female (red line).

Top right hand side: Comparison of central volume (V1) vs. different ages simulated by allometric scaling model of bevacizumab for male (blue line) and female (red line).

Bottom left hand side: Comparison of intercompartmental clearance versus different ages simulated by allometric scaling model of bevacizumab for male (blue line) and female (red line).

Bottom right hand side: Comparison of peripheral volume (V2) vs. different ages simulated by allometric scaling model of bevacizumab for male (blue line) and female (red line).

Table S4: Comparison of Model predicted (PBPK and Allometric Scaling) and observed PK parameters of bevacizumab

Dose [mg/kg]	PK Parameters	Observed[a)]	Predicted - PBPK, 13 years, male (with growth)	Predicted- Allometric Scaling 13 years, male
15	$AUC_{0\text{-}inf}$ [µg/ml*days]	3180 ± 1655	4894	4302
15	$T_{1/2}$ [days] (2 weeks)	9.6 ± 4.4	19.7	12.9
15	V_{ss} [ml/kg]	66.2 ± 15	77.6	60.9
5	$AUC_{0\text{-}inf}$ [µg/ml*days]	1115 ± 264	1475	1434
5	$T_{1/2}$ [days] (2 weeks)	11.9 ± 1.7	18.5	12.9
5	V_{ss} [ml/kg]	73.7 ± 8.3	71.7	60.9

a) Mean values and standard deviation reported in literature (Glade Bender et al., 2008). Age range of patients included in the study was 1-21 years, median age: 13 years.

7 References

Claassen, K., Thelen, K., Coboeken, K., Gaub, T., Lippert, J., Allegaert, K., and Willmann, S. (2015). Development of a Physiologically-Based Pharmacokinetic Model for Preterm Neonates: Evaluation with In Vivo Data. *Curr Pharm Des* 21**,** 5688-5698.

Glade Bender, J.L., Adamson, P.C., Reid, J.M., Xu, L., Baruchel, S., Shaked, Y., Kerbel, R.S., Cooney-Qualter, E.M., Stempak, D., Chen, H.X., Nelson, M.D., Krailo, M.D., Ingle, A.M., Blaney, S.M., Kandel, J.J., Yamashiro, D.J., and Children's Oncology Group, S. (2008). Phase I trial and pharmacokinetic study of bevacizumab in pediatric patients with refractory solid tumors: a Children's Oncology Group Study. *J Clin Oncol* 26**,** 399-405.

Gordon, M.S., Margolin, K., Talpaz, M., Sledge, G.W., Jr., Holmgren, E., Benjamin, R., Stalter, S., Shak, S., and Adelman, D. (2001). Phase I safety and pharmacokinetic study of recombinant human anti-vascular endothelial growth factor in patients with advanced cancer. *J Clin Oncol* 19**,** 843-850.

Han, K., Peyret, T., Quartino, A., Gosselin, N.H., Gururangan, S., Casanova, M., Merks, J.H., Massimino, M., Grill, J., Daw, N.C., Navid, F., Jin, J., and Allison, D.E. (2016). Bevacizumab dosing strategy in paediatric cancer patients based on population pharmacokinetic analysis with external validation. *Br J Clin Pharmacol* 81**,** 148-160.

Robbie, G.J., Zhao, L., Mondick, J., Losonsky, G., and Roskos, L.K. (2012). Population pharmacokinetics of palivizumab, a humanized anti-respiratory syncytial virus monoclonal antibody, in adults and children. *Antimicrob Agents Chemother* 56**,** 4927-4936.

Subramanian, K.N., Weisman, L.E., Rhodes, T., Ariagno, R., Sanchez, P.J., Steichen, J., Givner, L.B., Jennings, T.L., Top, F.H., Jr., Carlin, D., and Connor, E. (1998). Safety, tolerance and pharmacokinetics of a humanized monoclonal antibody to respiratory syncytial virus in premature infants and infants with bronchopulmonary dysplasia. MEDI-493 Study Group. *Pediatr Infect Dis J* 17**,** 110-115.

Valentin, J. (2002). Basic anatomical and physiological data for use in radiological protection: reference values: ICRP Publication 89. *Annals of the ICRP* 32**,** 1-277.
